# Synthesis, structural characterization, and antimicrobial evaluation of new mononuclear mixed ligand complexes based on furfural-type imine ligand, and 2,2′-bipyridine

**DOI:** 10.1038/s41598-023-36060-0

**Published:** 2023-06-06

**Authors:** Basma A. Ismail, Zeinab H. Abd El-Wahab, Omyma A. M. Ali, Doaa A. Nassar

**Affiliations:** 1 Chemistry Department, Faculty of Women for Arts, Science and Education, Ain Shams University, Cairo, Egypt; 2 Chemistry Department, Faculty of Science (Girl’s), Al-Azhar University, Youssif Abbas St., P.O. Box 11754, Nasr City, Cairo, Egypt

**Keywords:** Chemical biology, Chemistry

## Abstract

The present investigation goal was to investigate the chemistry of four new mononuclear mixed ligand Fe(III), Co(II), Cu(II), and Cd(II) complexes constructed from furfural-type imine ligand (L), and the co ligand 2,2′-bipyridine in addition to assessing their antimicrobial activity against some bacterial, and fungi strains. The structure of the complexes was interpreted by different spectroscopic techniques such as MS, IR, ^1^H NMR, UV–Vis, elemental analysis, TG-DTG, conductivity, and magnetic susceptibility measurements. The correlation of all results revealed that ligand (L) acts as a neutral ONNO tetradentate whereas the co ligand acts as a neutral NN bidentate. The coordination of the ligands with the metal ions in a molar ratio of 1:1:1 leads to formation of an octahedral geometry around the metal ions. The octahedral geometry has been validated and optimized by DFT analysis. Conductivity data showed the electrolytic nature of all complexes. The thermal stability of all complexes was deduced in addition to evaluating some thermodynamic, and kinetic parameters using Coats–Redfern method. Furthermore, all complexes in comparison to their parent ligands were tested for their biological potency against some pathogenic bacterial, and fungi strains using the paper disk diffusion method. [CdL(bpy)](NO_3_)_2_ complex revealed the highest antimicrobial activity.

## Introduction

Due to their ability to coordinate with different metal ions, forming stable complexes with attractive structural variance, Schiff bases as organic compounds represent a great class of characteristic ligands in coordination chemistry. Their corresponding complexes are characterized by remarkable structure, and significant geometry has adapted them to be useful for various applications in medicine, and pharmaceutical fields in addition to their potential applications in catalysis, material science, and others. Moreover, the physical, and chemical properties of Schiff bases metal frameworks in addition to their geometrical structures are affected by the nature of the ligand structure, and the metal ion as building blocks^1–3^. Hence, it is fundamental to select suitable ligands, and metal ions to discover new metal complexes that display beneficial properties with a diverse structure. In continuance of our previously achieved mixed ligand complexes^4–12^, the current research aims to synthesize new mixed-ligand complexes based on furfural-type imine ligand (L) as primary ligand, and 2,2′-bipyridine (2,2′-bpy) as a co-ligand in the presence of iron(III), cobalt(II), copper(II), and cadmium(II) ions. Selection of 2,2′-bpy to act as a co-ligand because it is an electron-conjugated heterocyclic aromatic ligand has a higher coordinating ability due to its *N*-donor chelation nature forming stable complexes^13^. Several studies have focused on the use of 2,2′-bpy as a co-ligand in mixed ligand complex synthesis revealing that 2,2′-bpy is a strong chelating ligand capable of strongly binding, in a bidentate manner to several types of metal ions forming very stable complexes with five-membered chelate rings through the two pyridine ring nitrogen atoms^14^. Furthermore, the mixed-ligand technique is pleasurable as a result of its design which allows different functional groups with variable binding sites, and tightly binds to the metal ion forming stable mixed-ligand complexes. These mixed-ligand complexes are beneficial for specific applications in inorganic chemistry, biology, biochemistry, medicine, physics, industry, and others^15^. The newly synthesized mixed ligand complexes have been structurally characterized by different techniques. Also, the optical band gap energy in addition to some other optical parameters has been calculated for the synthesized complexes. Besides, the molecular structure of the prepared complexes was investigated based on DFT calculations. The results of bioactivity test for antibacterial, and antifungal activities were also included.

## Experimental section

### Materials

All the chemicals used in this work were bought from Sigma Aldrich and used without further purification. The furfural-type imine ligand (L) {N^1^,N^2^-bis(furan-2-ylmethylene)-4-methylbenzene-1,2 diamine} was synthesized in our previous study^16^.

### Instrumentation and measurement

For all synthesized mixed ligand complexes, the elemental analysis (C, H, N) was carried out using a Perkin–Elmer-2400 CHN elemental analyzer, the chloride content was determined by gravimetric, and the metal content was estimated using complexometric titration after digesting their corresponding complexes with a 1:1 mixture ratio of concentrated H_2_SO_4_/HNO_3_ acids^17^. Infrared measurements were obtained as KBr plates using a Shimadzu 8000 FT–IR spectrometer. A Varian-Mercury 300 MHz spectrometer was used to record the ^1^H NMR spectrum of Cd(II) complex dissolved in (CD_3_)_2_SO, and the chemical shift values were recorded in the ppm unit. The UV–vis Shimadzu spectrophotometer (UV-2600) was utilized to obtain the electronic spectra of the complexes dissolved in DMSO at ambient temperature in the 200–700 nm range. The JEOL JMS-AX 500 spectrometer was used to record the mass of the complexes. Thermal analyses (TG / DTG) were recorded using a Shimadzu DT-50 thermal analyzer in a nitrogen atmosphere at heating rate of 10 °C/min up to 800 °C. Magnetic susceptibilities measurements were done using the Sherwood magnetic susceptibility balance at room temperature. The conductivity measurements were measured for the complexes dissolved in DMF (1 × 10^–3^ M) at ambient temperature using a Jenway 4010 conductivity meter. Melting points were determined using a Stuart Scientific SMP30 instrument.

### Mixed ligand complexes synthesis

**Figure 1:**
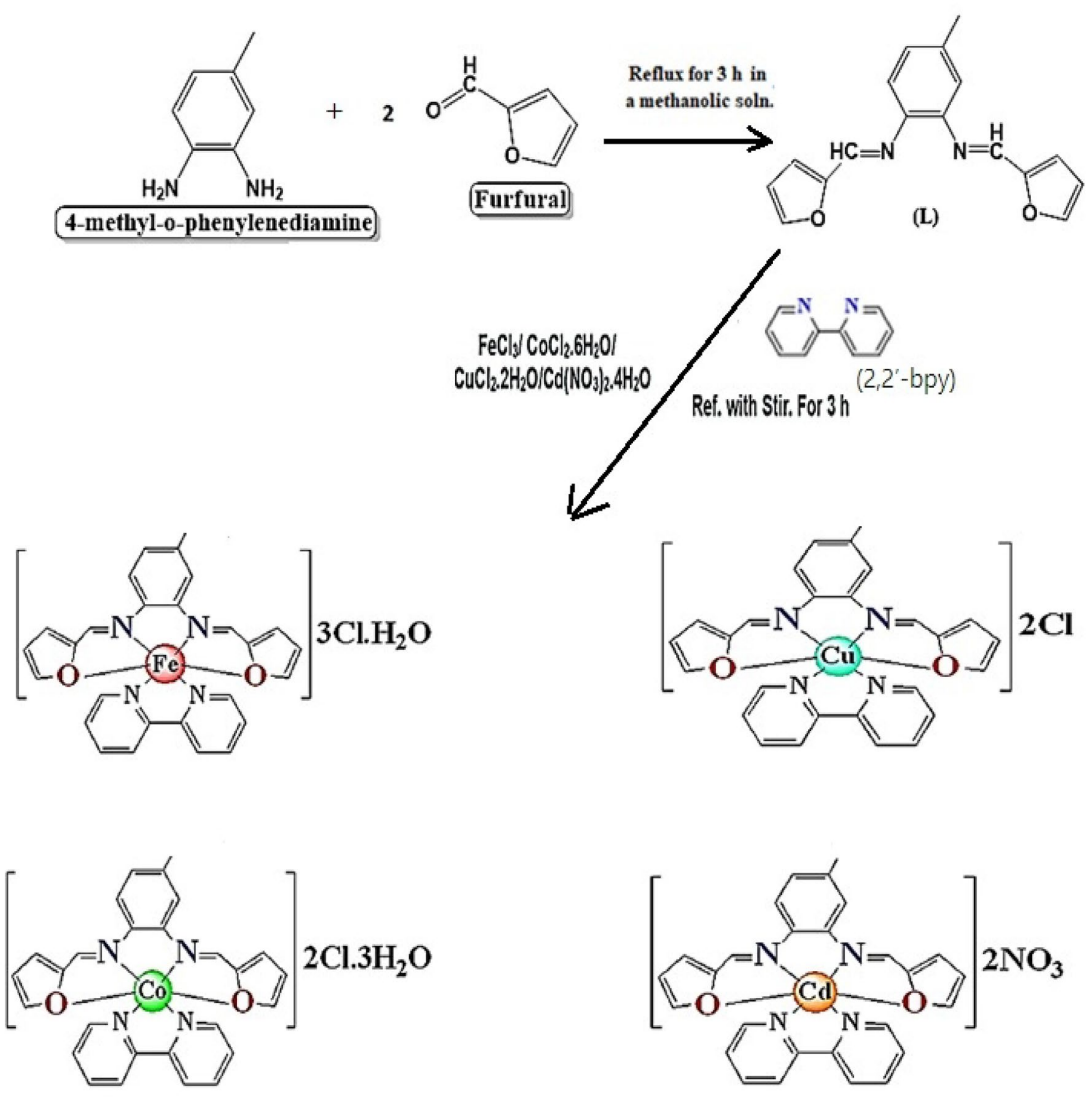
Synthetic procedure for the imine ligand (L), and its corresponding mixed ligand complexes with the co ligand (2,2′-bpy) along with their structures.

To synthesize the mixed ligand Fe(III), Co(II), Cu(II), and Cd(II) complexes, a methanolic solution of the co-ligand, 2,2′-bpy (0.16 g, 1 mmol) was added drop wise to a stirred solution of the imine ligand, L (0.28 g, 1 mmol) dissolved in methanol followed by adding dropwise 1 mmol of FeCl_3_ (0.16 g), CoCl_2_·6H_2_O (0.24 g), CuCl_2_·2H_2_O (0.17 g), and Cd(NO_3_)_2_·6H_2_O (0.29 g) salts dissolved in the same solvent separately with constant stirring. Thereafter, the resulting reaction mixture was refluxed with continuous stirring for 3 h. During this time, a complete dissolution of the reaction mixture was noticed accompanied by precipitate formation. The formed solid complexes were filtered off, washed with methanol followed by diethyl ether, and finally dried under vacuum, and kept under CaCl_2_-dryness in a desiccator. The synthetic procedure for the imine ligand (L), and its corresponding mixed ligand.complexes with 2,2′-bpy along with their structures are shown in Fig. 1.

### Theoretical calculations

Density functional theory (DFT) optimization geometry and frontier molecular orbitals of the complexes were calculated using Gaussian 09 software package. B3LYP functional was used with 6-311G++ (d,p) basis set for C, H, N, O, Cl and LANL2DZ for complexes in gas phase^18^.

### In vitro antimicrobial assay

Antimicrobial assays of all prepared mixed ligand complexes were investigated against different bacterial strains, such as *Staphylococcus aureus* (**+ **ve), *Bacillus subtilis* (**+ **ve), *Proteus vulgaris* **(-**ve), *Escherichia coli* (**− **ve). Furthermore, antifungal screening was studied against two strains, *Aspergillus flavus*, and *Candida albicans*. The standard antibiotics used in bacteria and fungi tests were *Gentamicin*, and *Ketoconazole*, respectively. The method of paper disk diffusion was used for the determination of the antimicrobial activities of each microorganism in which Mueller Hinton-agar, and malt agar medium were applied to bacterial and fungal studies, respectively^19^. The tested compounds were dissolved in a minimal volume of DMSO to receive concentrations of 1 mg/mL, and 100 μl of each result was used. The inhibition zone values were calculated in millimeters, and the results were taken as the mean of triplicate. Additionally, DMSO was subjected to the same experiment under similar conditions, and no activity was detected.

### Ethical approval

The manuscript does not include any studies on human or animals.

## Results and discussion

The mixed ligand Fe(III), Co(II), Cu(II), and Cd(II) complexes were prepared by mixing equimolar amounts of the furfural-type imine ligand (L), bipyridine (2,2′-bpy) as co-ligand, and FeCl_3_, CoCl_2_.6H_2_O, CuCl_2_.2H_2_O, and Cd(NO_3_)_2_.6H_2_O solutions separately in a good yield ranging from 82 to 95%. All isolated complexes were stable in air, and ambient temperature, and characterized by interesting color, and a high melting point. Additionally, they are insoluble in water, and common organic solvents except DMF and DMSO solvents.

### Elemental composition analysis

Based on the elemental analysis data (Table 1), the mononuclear mode with 1:1:1 stoichiometry (L:bpy:M) of all metal complexes was first suggested. The observed elemental data (C, H, N, Cl, and metal ions) were exactly matched with the calculated ones, and provide the actual construction of the prepared complexes. Some information such as the empirical formula, molecular weight, color, yield percentage, and molar conductance of the isolated complexes is depicted in Table 1. The equations that describe the complex's formation are represented as:

$$\begin{gathered} {\text{L}} + {\text{bpyMX}} \cdot {\text{yH}}_{{2}} {\text{O}} \to \left[ {{\text{ML}}\left( {{\text{bpy}}} \right)} \right]{\text{Z}} + {\text{nH}}_{{2}} {\text{O}} \hfill \\ {\text{M}} = {\text{ Fe}}\left( {{\text{III}}} \right),{\text{ X}} = {\text{Cl}}_{{3}} ,{\text{ y}} = 0,{\text{ Z}} = {\text{Cl}}_{{3}} .{\text{H}}_{{2}} {\text{O}},{\text{ n}} = 0 \hfill \\ {\text{Co}}\left( {{\text{II}}} \right),{\text{ X}} = {\text{Cl}}_{{2}} ,{\text{ y}} = {6},{\text{ Z}} = {\text{Cl}}_{{2}} .{\text{3H}}_{{2}} {\text{O}},{\text{ n}} = {3} \hfill \\ {\text{Cu}}\left( {{\text{II}}} \right),{\text{ X}} = {\text{Cl}}_{{2}} ,{\text{ y}} = {2},{\text{ Z}} = {\text{Cl}}_{{2}} ,{\text{ n}} = {2} \hfill \\ {\text{Cd}}\left( {{\text{II}}} \right),{\text{ X}} = \left( {{\text{NO}}_{{3}} } \right)_{{2}} ,{\text{ y}} = {6},{\text{ Z}} = \left( {{\text{NO}}_{{3}} } \right)_{{2}} ,{\text{ n}} = {6} \hfill \\ \end{gathered}$$

**Table 1:** Analytical and some physical data of the synthesized mixed ligand complexes.

Mixed ligand complexes	M.Wt Found. (Calcd)	Color (% yield)	Elemental analysis, Found (Calcd.)%						Molar conductance (Ω^−1^ mol^−1^cm^2^)
			C	H	N	Cl	M	H_2_O	
[FeL(bpy)]Cl_3_·H_2_O FeC_27_H_22_N_4_O_2_Cl_3_.H_2_O	615.00 (614.72)	Dark red (95)	52.81 (52.76)	3.28 (3.94)	9.54 (9.11)	17.01 (17.30)	8.97 (9.08)	2.10 (2.00)	280
[CoL(bpy)]Cl_2_·3H_2_O CoC_27_H_22_N_4_O_2_Cl_2_·3H_2_O	621.00 (618.38)	Dark green (95)	51.99 (52.44)	4.48 (4.56)	8.91 (9.06)	11.21 (11.46)	9.43 (9.52)	8.70 (8.73)	163
[CuL(bpy)]Cl_2_ CuC_27_H_22_N_4_O_2_Cl_2_	570.00 (568.95)	Faint green (96)	56.91 (57.00)	3.55 (3.90)	9.37 (9.85)	12.30 (12.46)	11.01(11.15)	–	143
[CdL(bpy)](NO_3_)_2_ CdC_27_H_22_N_6_O_8_	670.00 (670.92)	Cacao brown (82)	47.68 (48.34)	3.53 (3.31)	12.75 (12.53)	–	16.34 (16.75)	–	176

### Mass spectral studies

**Figure 2:**
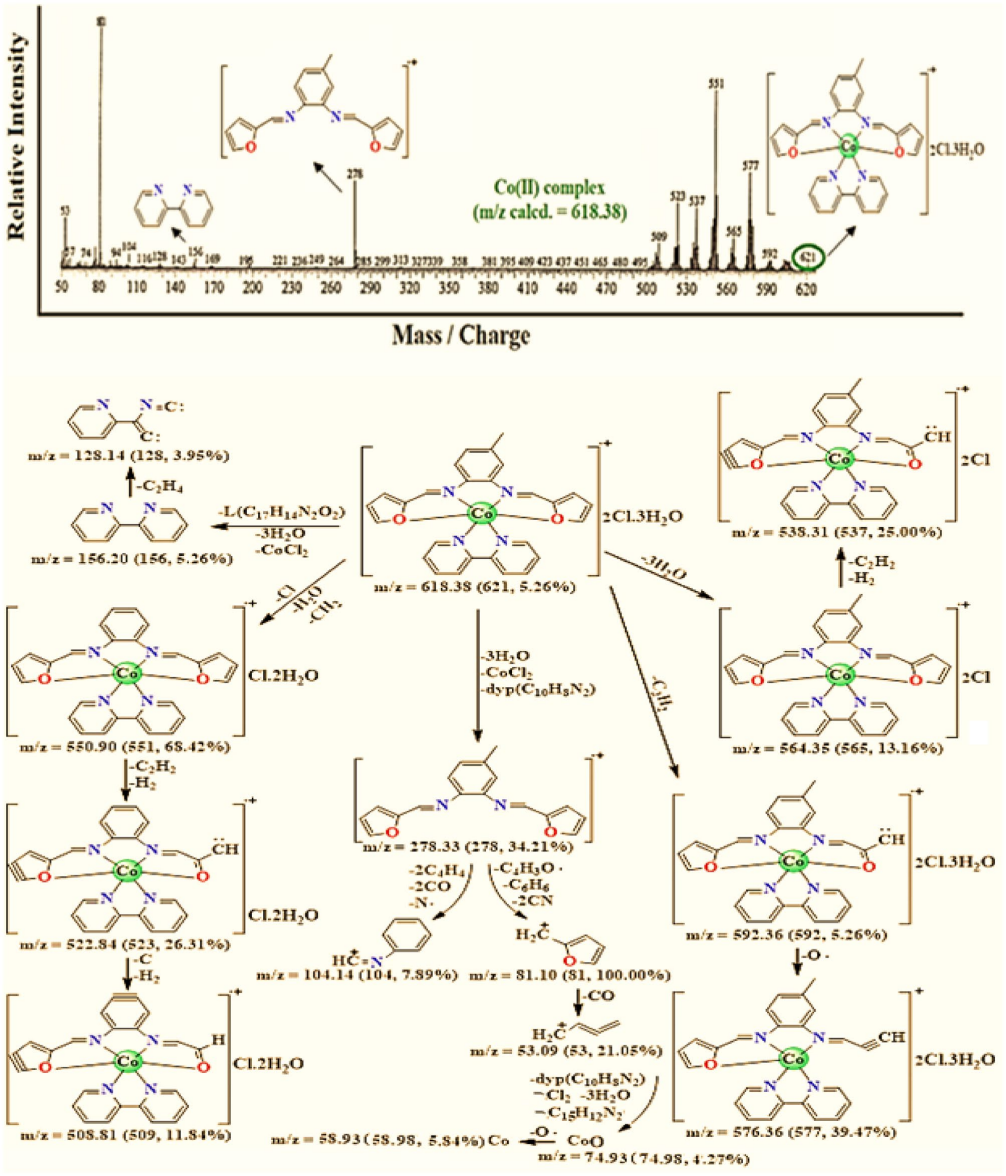
Mass spectrum, and mass fragmentation of [CoL(bpy)]Cl_2_.3H_2_O complex {The data under each fragment represent *m*/*z* calculated (found, intensity)}.

The structural features of the mixed ligand Fe(III), Co(II), Cu(II), and Cd(II) complexes were estimated based on the mass spectral analysis. For all prepared complexes, the molecular ion peaks with m/z emit at Fe(III) 615.00, Co(II) 621.00, Cu(II) 570.00, and Cd(II) 670.00. These data showed good agreement with the calculated values (Table 1) utilizing the elemental analyses as 614.72, 618.38, 568.95, and 670.92, respectively. Consider the mass spectrum, and fragmentation pattern of the mixed ligand Co(II) complex as a representative example (Fig. 2), the presence of the molecular peaks at *m*/*z* 278.00; 34.21% (Calcd. 278.33), and *m*/*z* 156.00; 5.26% (Calcd. 156.20) that correspond to the ligand moiety (L; C_17_H_14_N_2_O_2_), and 2,2′-bipyridine moiety (2,2′-bpy; C_10_H_8_N_2_), respectively confirm the proposed structure, and the successful preparation of the complexes.

### IR spectra studies

**Figure 3:**
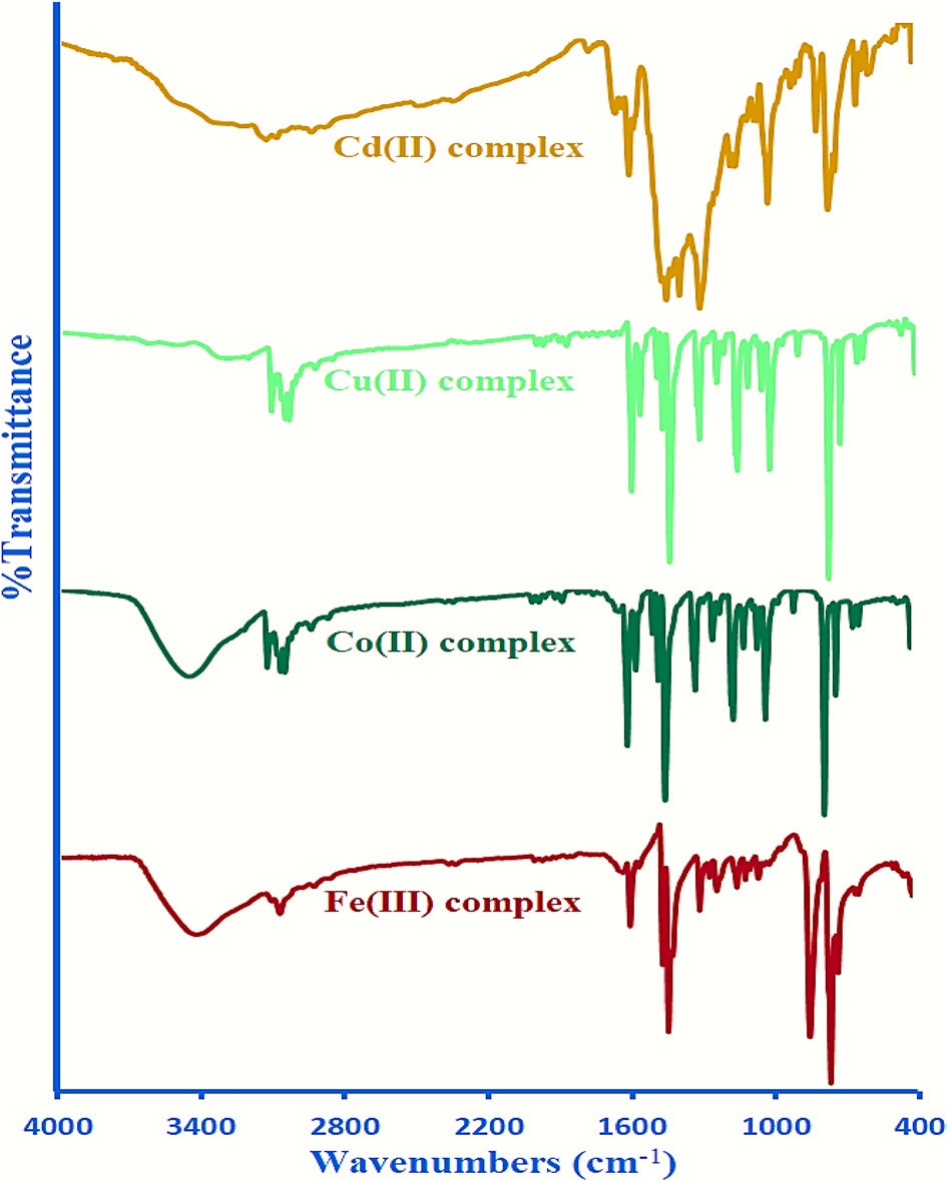
IR spectra of the mixed ligand complexes.

The IR spectra of the four synthesized mixed ligand complexes, in addition to the imine ligand (L), and 2,2′-bpy are listed in Table 2. Two intense absorption peaks at 1602–1598, and 1309–1286 cm^-1^ observed in the IR spectrum of the mixed ligand complexes (Fig. 3) could be ascribed to the imine group ν(C=N), and furanyl ring moiety ν(C–O–C), respectively. The observed negative, and positive shifts by 12–16, and 2–25 cm^-1^ for ν(C=N), and ν(C–O–C) of the complexes as compared to 1614, and 1284 cm^-1^, respectively, of the free imine ligand (L) clearly point to the participation of azomethine nitrogen^20^, and furanyl oxygen atoms in coordination with the metal ions^21^. Furthermore, the characteristic peak of C=N associated with the free 2,2′-bpy co-ligand located at 1553 cm^-1^ showed that all four synthesized mixed ligand complexes in a range of 1587–1567 cm^-1^ agree with the pyridyl nitrogen atoms chelation around the metal ions^22^. In addition, all synthesized mixed ligand complexes showed new peaks in the range of 596–472 cm^−1^ refer to M–N, and M–O vibrations that confirmed the assumption of the coordination sites about the metal ions^23^. Only, the two Fe(III), and Co(II) mixed ligand complexes show broad bands at about 3411 and 3440 cm^-1^, respectively, in their IR spectra referred to as the ν(OH_2_) stretching vibration mode for water molecules^24^. This assumption is compatible with the elemental analysis results and confirmed by thermal analysis as given later. Also, the Cd(II) complex spectrum showed a characteristic frequency for the ionic nitrate as an intense peak appeared at 1384 cm^−1^^25^ confirmed by the conductance measurements. These findings, and the changes in profile of the stretching frequencies for the four synthesized mixed ligand complexes as compared with those observed for the ligand, L, and 2,2′-bipyridine co-ligand confirm the chelation sites of the ligands with the metal ions, and the formation of metal–ligand bonds through these sites, which are the possible binding sites for the coordination.

**Table 2:** IR spectral (cm^−1^), and ^1^H-NMR (δ, ppm) data of the imine ligand (L), the co-ligand (bpy), and its mixed ligand complexes.

Compound under study	IR spectra (cm^−1^)								^1^H-NMR (δ, ppm)
	ν(OH_2_)	ν(C = N) azomethine / pyridyl		ν(C–O–C) (furan)	δ(py) (bending)	υ(M–O)	υ(M–N)	Additional bands	
L	–	1614	–	1284	–	–	–	–	s (8.47, HC = N); m (8.12–8.10, 3H–fur., *J* = 8; 7.03–7.01, 3H–fur., *J* = 10; 5.89–5.85, 3H–arm., *J* = 16); 2.56 (s, C–CH_3_)
2,2′-bipyridine	–	–	1553	–	660	–	–	–	
[FeL(bpy)]Cl_3_·H_2_O	3411	1602	1569	1309	648	582	481	–	–
[CoL(bpy)]Cl_2_·3H_2_O	3440	1601	1568	1289	651	580	495	–	–
[CuL(bpy)]Cl_2_	–	1602	1567	1286	659	580	484	–	–
[CdL(bpy)](NO_3_)_2_	–	1598	1587	1299	649	596	497	1384; νNO_3_ ionic	s (8.99, HC = N); m (7.64–7.61, 3H–fur., *J* = 10; 7.15–7.12, 3H–fur., *J* = 11.2; 5.73–5.72, 3H–arm., *J* = 5.2); 2.72 (s, C–CH_3_)

### ^1^H NMR studies

**Figure 4:**
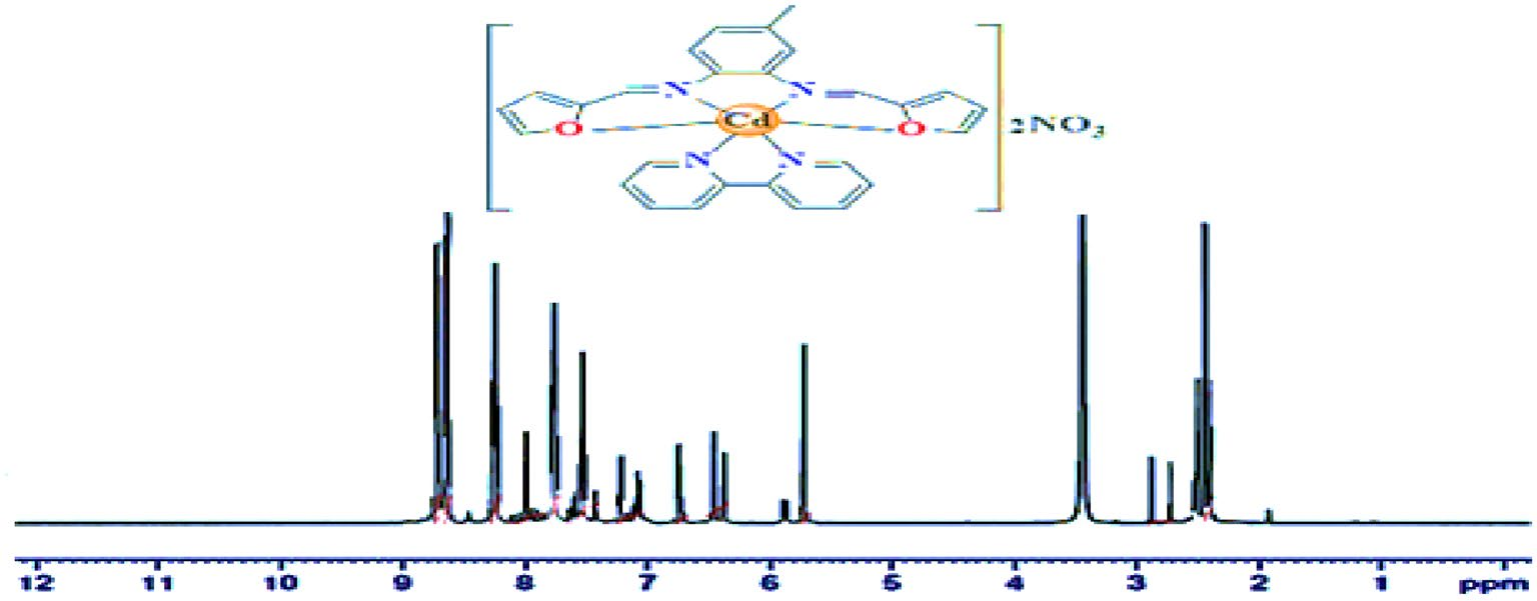
^1^H NMR spectrum of [CdL(bpy)](NO_3_)_2_ complex.

In the ^1^H NMR spectrum of the diamagnetic Cd(II) complex (Fig. 4), two singlet signals were observed at δ 2.72, and 8.99 ppm in comparison to δ 2.56, and 8.47 ppm in the ligand L spectrum. The first signal is due to the methyl protons, whereas the second one is due to the azomethein proton (–CH=N–)^26,27^. The observed higher chemical shift value of the signal due to the azomethein proton affirms the bonding of the ligand L, to the Cd(II) ion through the imine nitrogen atom. Additionally, the ligand L, and Cd(II) complex reveal some multiplet signals due to phenyl, furanyl, and pyridyl rings protons that were well established in their predictable regions as given in Table 2 with their coupling constant *J* (Hz)^28,29^.

### Conductance behavior study

The molar conductance measurements for the synthesized mixed ligand complexes were performed at ambient temperature on a freshly prepared solution of each metal complex in DMF as a solvent at a concentration of 1 × 10^–3^ M. The obtained conductance data establish the ionic nature of the synthesized mixed ligand complexes as a result to the presence of chloride or nitrate anions outside the coordination sphere. The obtained measurement values (Table 1) for the mixed ligand Co(II), Cu(II), and Cd(II) complexes, were in the range of 143–176 Ω^−1^ mol^−1^ cm^2^ whereas for the Fe(III) complex, the value was 280 Ω^−1^ mol^−1^ cm^2^ indicating that all complexes are electrolytic types. These values show a 1:3 electrolytic nature for the Fe(III) complex, and an electrolytic nature of the type 1:2 for other complexes^30^. Additionally, the presence of Cl^−^ ion outside the coordination sphere is confirmed by a white precipitate formation upon addition of AgNO_3_ solution^31^. These results are also supported by comparing them with similar work elsewhere and confirm the assumption that the nonparticipation of the counter anions in the coordination sphere is about the metal ions.

### Magnetic susceptibility measurements

The measurements of the magnetic susceptibility for the metal complexes were done at room temperature to estimate the effective magnetic moment value (µ_eff_) that help in the geometrical structural investigation via the determination of the number of unpaired electrons. Magnetic susceptibility measurements for all complexes, Table 3 display their paramagnetic properties except for the Cd(II) complex. For the Fe(III) complex, the estimated µ_eff_ was 5.97 B.M which is near the theoretical value of 5.92 B.M indicating a high-spin complex with five unpaired electrons, and octahedral geometry^32^. The Co(II) complex has a µ_eff_ value of 4.31 B.M. This value arises from the existence of three unpaired electrons but it is noticeably higher than those described for the spin only value (3.87 B.M), and can be attributed to *d*^7^- system with an orbital angular momentum contribution^33^. The estimated µ_eff_ value for the Cu(II) complex was 1.65 B.M which is in compliance with the Cu(II) ion containing one unpaired electron, and near the theoretical value (1.73 B.M) corresponding to one unpaired electron^34^. The magnetic susceptibility measurement for the Cd(II) complex showed its diamagnetic character, which illustrates that it has no unpaired electron^35^.

**Table 3:** Magnetic properties, and electronic spectra data of the synthesized mixed ligand complexes.

Mixed ligand complexes	Magnetic moment values (B.M)				UV–Vis spectrophotometer data λ_max_ (nm)				ligand field parameters			
	µ_eff_	µ_SO_	*n*(theory)	Hybrid orbitals	π → π	n → π	CTI	d → d transition/peak assignment	*D*q (cm^−1^)	*B* (cm^−1^)	*Β/β* %	LFSE (cm^−1^)
[FeL(bpy)]Cl_3_.H_2_O	5.97	5.92	5	*sp*^3^*d*^2^	248 286	327 333	379 386	494; ^6^A_1g_ → ^4^T_1g_ 534; ^6^A_1g_ → ^4^T_2g_	–	–	–	–
[CoL(bpy)]Cl_2_.3H_2_O	4.31	3.87	3	*sp*^3^*d*^2^	258 296	323 343	375 383	571; ^4^T_1g_(F) → ^4^A_2g_(F) 628; ^4^T_1g_(F) → ^4^T_1g_(P)	1005	738	0.66 /34.11	8.04 × 10^3^
[CuL(bpy)]Cl_2_	1.65	1.73	1	*sp*^3^*d*^2^	251 300	307 328	361 372	707; ^2^B_1g_ → ^2^A_1g_ ^2^B_1g_ → ^2^E_g_	–	–	–	–
[CdL(bpy)](NO_3_)_2_	Diamagnetic				259 288	314 339	368 384	–	–	–	–	–

### UV–vis spectral study

The recorded electronic spectra for all mixed ligand complexes dissolved in DMF solution on a wavelength from 200 to 800 nm show several absorption peaks, and their assignments were made by comparing them with those reported in the literature. The observed peaks, Table 3 at a range of 248–300 nm assigned to the transition π → π* due to the presence of π-electrons in the conjugated system of phenyl, furanyl, and pyridyl rings as well as the C=N chromophore connected with imine, and pyridyl moieties. Also, the observed peaks in the range of 307–343 nm can be assigned to the n → π* transition as a result of the presence of the nonbonding electrons of the imine nitrogen, pyridyl nitrogen, and furanyl oxygen atoms functionalities. Moreover, the peaks at a range of 361–386 nm are consistent with intramolecular charge transfer^36^. All of this gives an indication of the chelation nature of the furfural-type imine ligand (L), and bipyridine with the metal ions. The electronic absorption spectrum of the Fe(III) mixed ligand complex exhibited two weak absorption peaks at 494, and 534 nm attributed to the two transitions ^6^A_1g_ → ^4^T_1g_, and ^6^A_1g_ → ^4^T_2g_, respectively. In accordance with that, the high spin octahedral arrangement of the Fe(III) cation characterized by the ^6^A_1g_ ground term and a series of weak transitions^37^. The spectrum of the Co(II) mixed ligand complex exhibited two absorption peaks at 571, and 628 nm connected with ^4^T_1g_(F) → ^4^A_2g_(F), and ^4^T_1g_(F) → ^4^T_1g_(P) transitions, respectively, suggesting an octahedral shape around the Co(II) ion^38^. Moreover, the ligand field parameters; (*B*), (*D*_q_), (*β*), (*β* %), and (LFSE) have also been directly evaluated for this complex from the spectra data, and are summarized in Table 3. The obtained results reveal that, the *B*- value (Racah interelectronic repulsion) is smaller than that reported for the free transition metal ion (1120 cm^−1^) suggesting a remarkable orbital overlap, and delocalization of metal *d*- electrons onto the ligands. Further, *β*- value (nephelauxetic ratio) is less than unity assuming a partial covalent metal ligand bonding^39^. The recorded spectrum for the Cu(II) mixed ligand complex displayed a low intensity broad peaks at 707 nm assigned to ^2^B_1g_ → ^2^A_1g_, and ^2^B_1g_ → ^2^E_g_ transitions suggest a distorted octahedral shape^40^. The Cd(II) complex spectrum does not furnish any *d*-*d* transition due to its completely filled *d*^10^ orbital.

### Optical properties for the mixed ligand complexes

Band gap energy (E_g_, eV) is the energy magnitude required for excitation of an electron between the valence, and conduction bands. Also, it describes the difference in energy between the top, and the bottom of these two bands. Estimation of the band gap energy of the materials is important to predict the potential use and performance of such materials in technologies, especially in optoelectronic applications. Additionally, UV–VIS absorption spectra are used to determine band gap energy^41,42^.

Actually, to determine the E_g_ value of the synthesized mixed ligand complexes, sequence steps are required. The absorption coefficient (α, cm^−1^) is evaluated at first using the data of absorbance (A) and thickness (t) as given in Eq. (1)^43^.

1 $${\alpha } = \frac{2.303\left( A \right)}{t}$$

**Figure 5:**
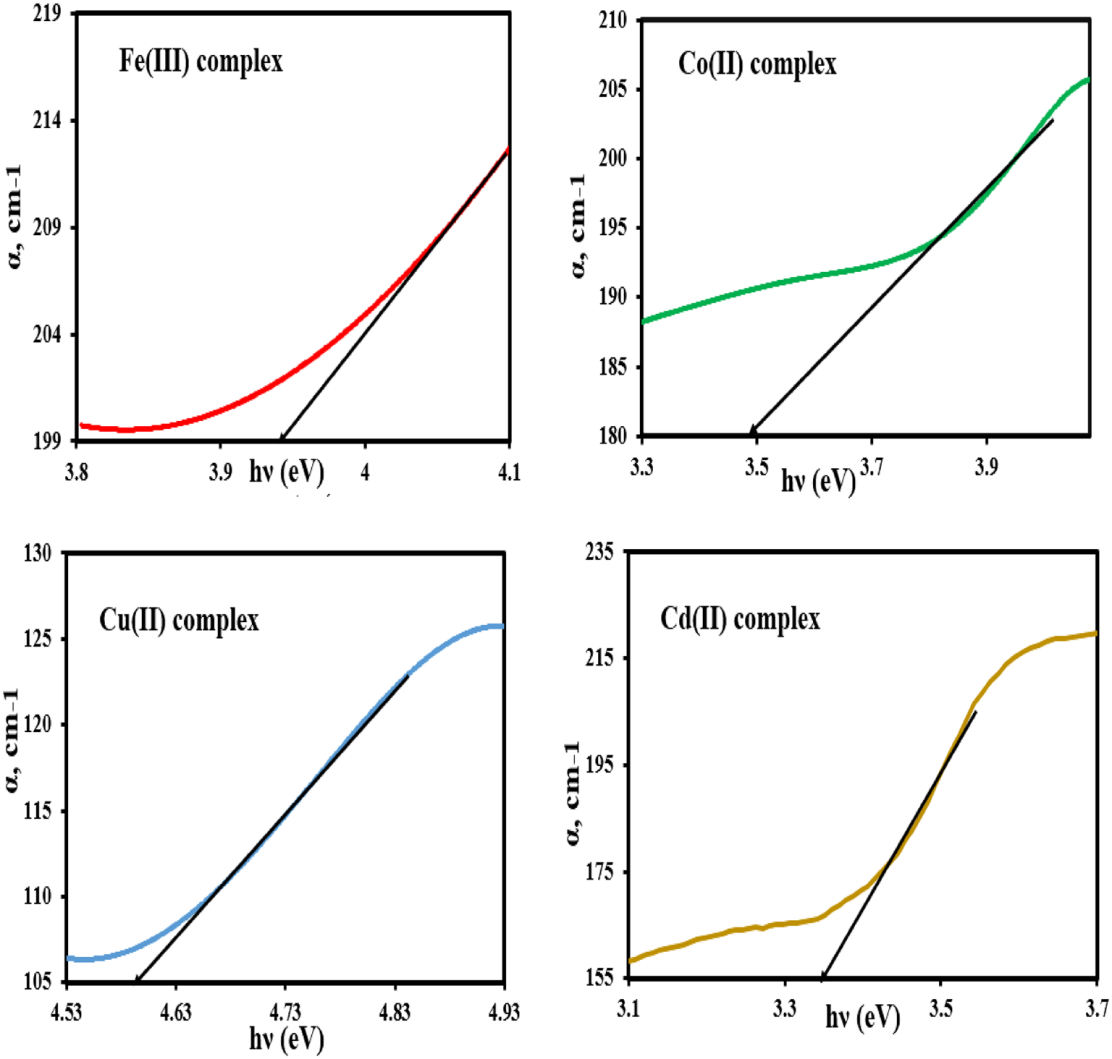
Plotting of absorption coefficient (α) versus photon energy (h*ν*) of the mixed ligand complexes.

The dependence of (α) values on the photon energy (hv) of the investigated complexes is presented graphically in Fig. 5. The fundamental absorption edge (E^e^, eV) of the investigated mixed ligand complexes was obtained by extrapolating the linear region in (α)—(hv) plot to the x- axis of the curve (α = zero)^44^. The values of E^e^ of the investigated complexes were found to be 3.94, 3.46, 4.59, and 3.34 eV for iron(III), cobalt(II), cupper(II), and cadmium(II) mixed ligand complexes, respectively, and listed in Table 4. Continuously, the calculated values of (α) are used to construct Tauc’s plot to obtain the band gap energy (E_g_) of the investigated complexes.

**Table 4:** Optical band-gap energy and some optical parameters of mixed ligand complexes.

Mixed ligand complexes	Tauc’s relation		ASF method		E^e^ (eV)	E_u_ (eV)	Parameters	
	E_g_^d^ (eV)	E_g_^i^ (eV)	*E*_g_^ASF^ (eV)	*E*_g_^ASF^ (eV)			*n*	σ_**s**_
[FeL(bpy)]Cl_3_·H_2_O	3.65	2.39	3.41	2.39	3.94	0.25	2.22	0.09
[CoL(bpy)]Cl_2_·3H_2_O	3.21	2.6	3.22	2.60	3.46	0.23	2.35	0.10
[CuL(bpy)]Cl_2_	4.52	2.98	4.53	2.98	4.59	0.81	2.06	0.02
[CdL(bpy)](NO_3_)_2_	3.10	3.27	3.14	3.25	3.34	0.24	2.38	0.09

In Tauc’s relation (Eq. 2), the absorption coefficient (*α*) is correlated with energy band gap (*E*_g_)^45^.

2 $$\upalpha hv = B\left( {hv - {\text{E}}_{{\text{g}}} } \right)^{n}$$

where (h), (*ʋ*), and (*B)* are Planck's constant, incident light frequency, and proportionality constant, whereas (*n*) is the optical frequency that expresses the nature of the transition process to be direct or indirect as: *n* = 1*/*2, and 2 for direct, and indirect allowed transitions, and *n* = 3*/*2, and 3 for direct, and indirect forbidden transitions, respectively.

**Figure 6:**
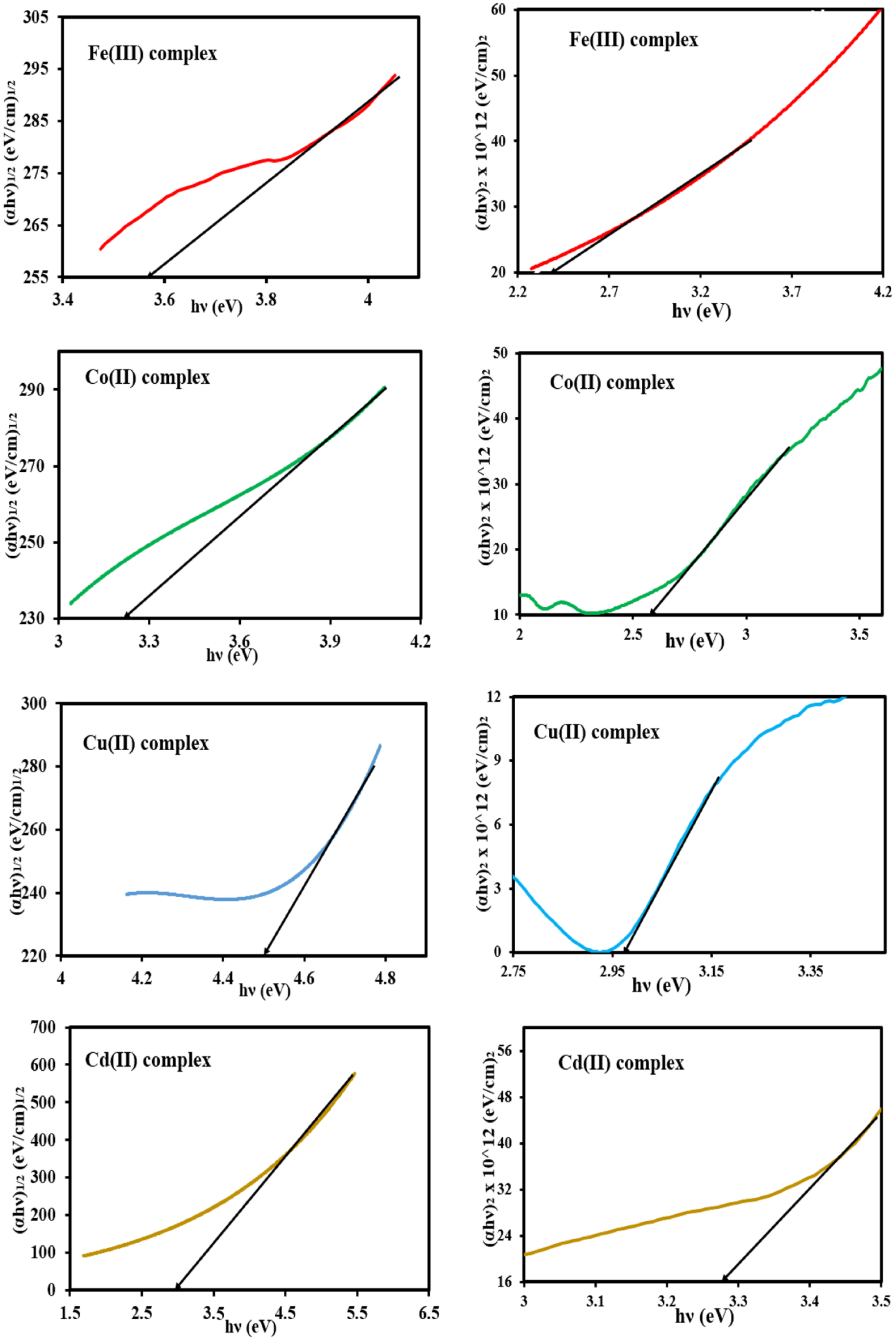
Plotting of (*α*h*ν*)^1/2^ and (*α*h*ν*)^2^ versus photon energy (h*ν*) for the mixed ligand complexes.

Tauc’s module is drawn as shown in Fig. 6 in which the incident photon energy (hν) along the x-axis is plotted against (αhν)^n^ along the Y-axis for the different possible values of n = 1/2, and 2. In continuation, by extending the linear portion of the graph onto the photon energy axis, the direct (E^d^_g_), and indirect (E^i^_g_) energy band gaps were determined at the cut-off point^46^. The obtained values of (E^d^_g_), and (E^i^_g_) energy band gap are summarized in Table 4.

Comparison of the fundamental absorption edge (E^e^) values with the direct (E^d^_g_), and indirect (E^i^_g_) energy band gap values, the direct allowed transitions were suggested for all synthesized mixed ligand complexes.

**Figure 7:**
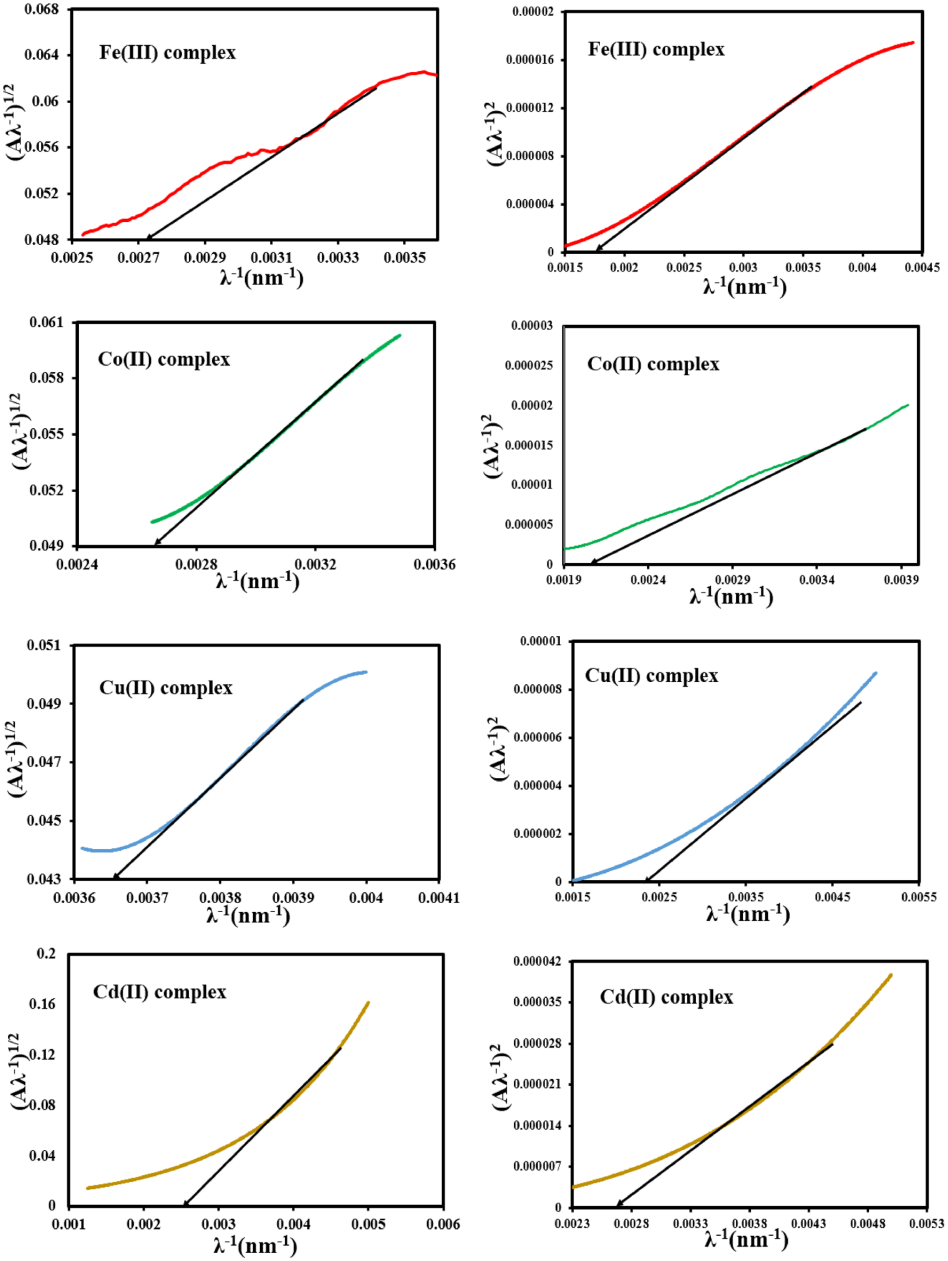
Relation between (A*λ*^−1^)^1/2^ and (A*λ*^−1^)^2^ vs. (*λ*^−1^) for the mixed ligand complexes.

A literature survey shows that, an accurate determination of the energy band gap can be achieved using the absorption spectra fitting (ASF) method in which the parameter (Aλ^−1^)^1/n^ along the y-axis is plotted against the parameter (λ^−1^) along the x-axis for the different possible values of n = 1/2, and 2 followed by a linear portion extrapolation of the graph onto the x-axis, Fig. 7. After that, *λ*_*g*_ values were determined from the cut-off point. So, the energy band gap values (*E*_g_^ASF^) of the studied complexes, Table 4 can be deduced using Eq. (3)^47^.

3 $$E_{g}^{ASF} = \frac{1239.83}{{{\uplambda }_{g} }}$$

**Figure 8:**
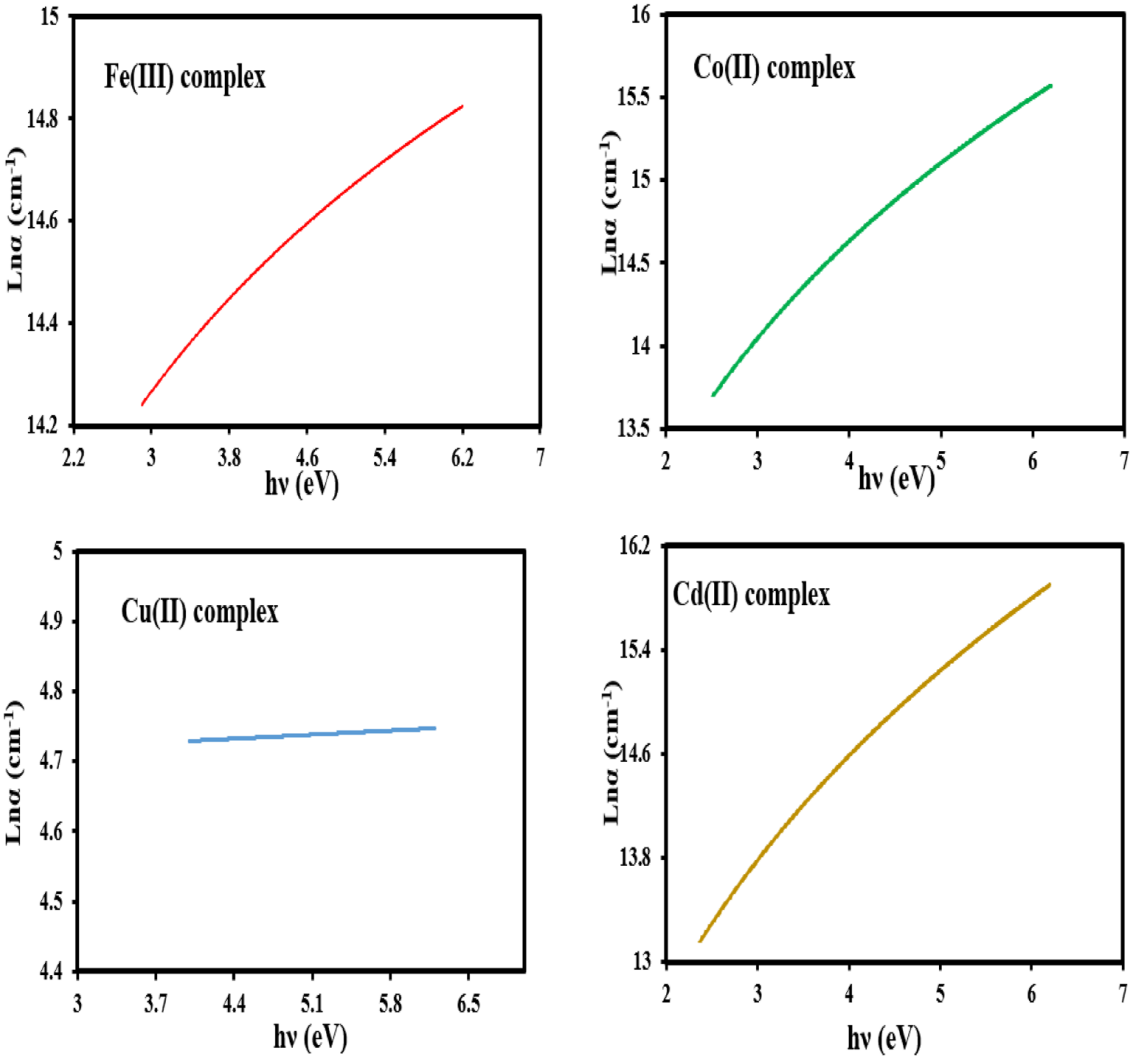
The Urbach plot ln (α) vs the photon energy (h*ν*) for the mixed ligand complexes.

Additionally, plotting of ln(α) versus *hv* (Fig. 8) gives a straight line. The inverse value of the slope for the straight line gives the value of Urbach energy (E_U_, eV), Table 4. The Urbach energy value gives information about the defects that may have originated during the preparation process^48^.

As clear from Table 4, the complexes can serve as semiconductor materials with band gap energy in the range of semiconductor materials^49^.

After all these, some optical parameters for the synthesized iron(III), cobalt(II), cupper(II), and cadmium(II) mixed ligand complexes can be investigated, and listed in Table 4 as the extinction coefficient (*k*), refractive index (*n*), and steepness coefficient using the fundamentally Eqs. (4–6)^50,51^.

4 $$k{ } = \frac{{{\upalpha }\lambda }}{4\pi }$$

5 $$en{ } = \frac{36.30}{{E_{g}^{{\left( {d,i} \right)}} }}$$

6 $$\sigma {\text{s }} = \frac{{k_{B} T}}{{E_{U} }}$$

**Figure 9:**
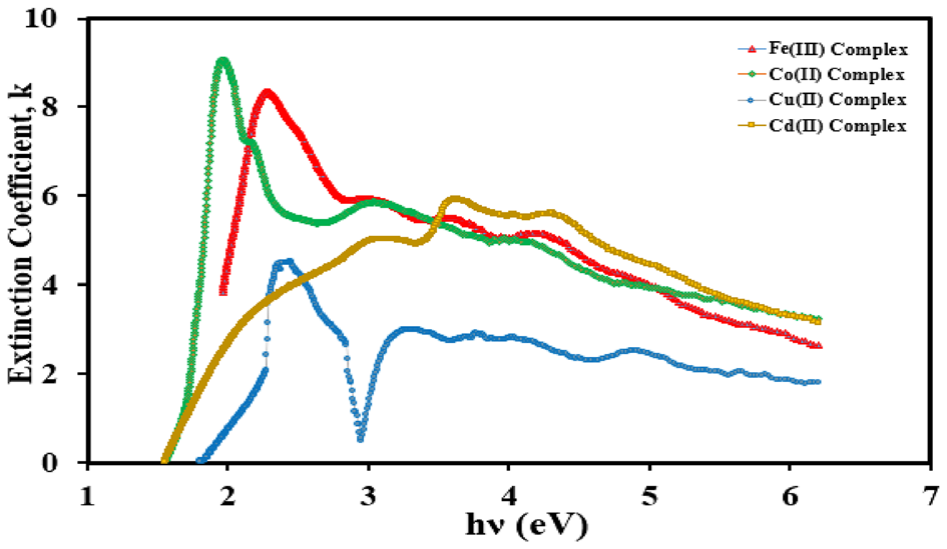
Variation of the extinction coefficient (*k*) with the wavelength (*λ*) for the mixed ligand complexes.

The variation of the extinction coefficient (*k*) with the wavelength (*λ*) for all complexes was represented graphically in Fig. 9. The value of (*k*) is increased with the increase of (*λ*) describing the increase in scattering rate for all complexes.

### Thermal analyses

**Figure 10:**
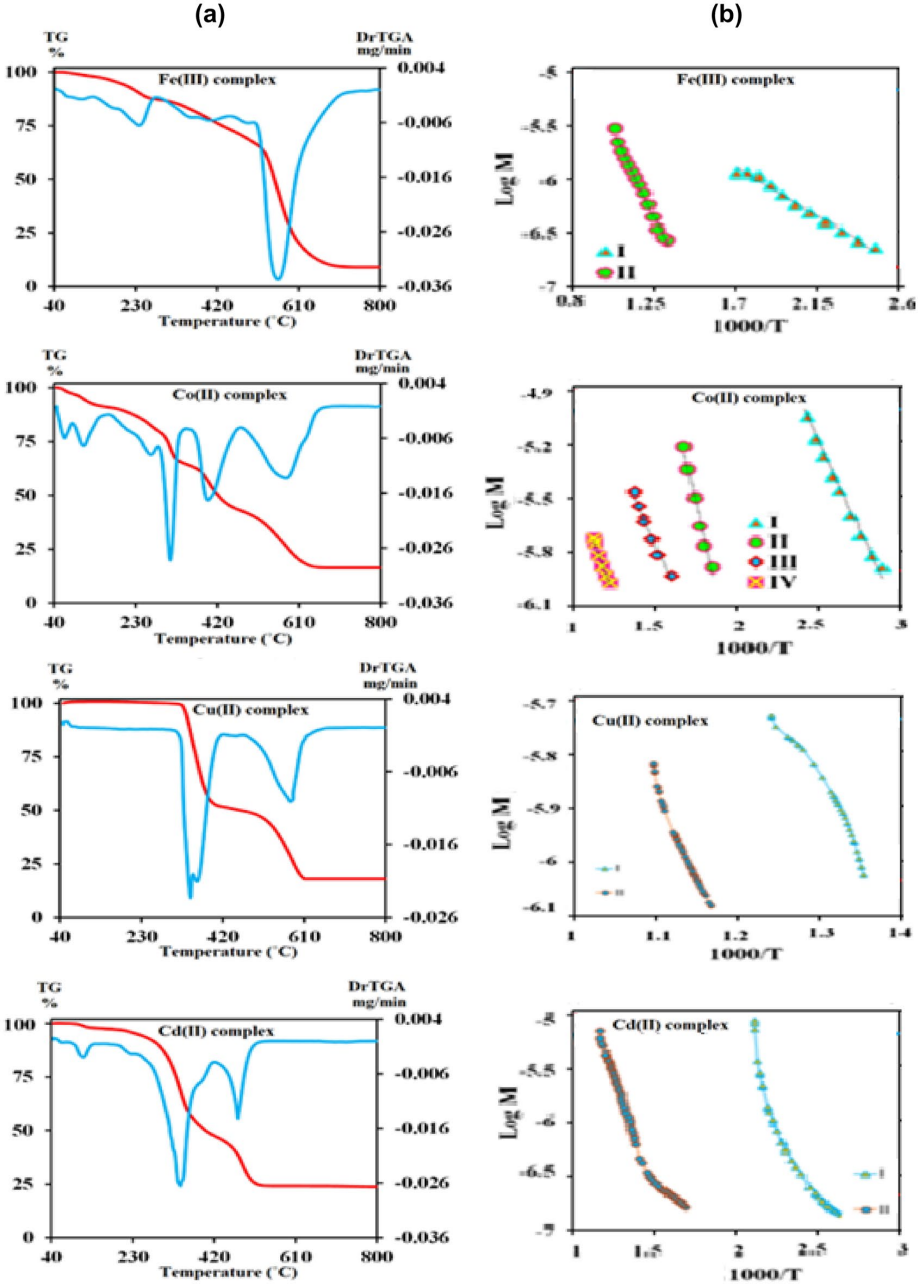
(**a**) TG-DTG thermograms of the mixed ligand complexes. (**b**) Coats-Redfern plots of mixed ligand complexes.

The thermal analysis study is the main study to detect the thermal stability of the synthesized metal complexes, and to investigate their structural building where, the water molecules nature; coordinated/hydrated, and the metal content is achieved through their mass decomposition^52^. The decomposition steps, temperature range, mass loss (%), mass loss assignments, leaving species, and the final residue are listed in Table 5, and TG-DTG thermograms of the prepared complexes are depicted in Fig. 10a.

**Table 5:** Thermogravimetric data of the mixed ligand complexes.

Mixed ligand complexes	Stage	Temp. range (°C)	Mass loss (%)		Evolved moiety	Residue (%) found (Calc.)
			Calc	Found		
[FeL(bpy)]Cl_3_·H_2_O	I	45–388	20.23	20.27	H_2_O(hyd.) and 1½Cl_2_	FeC_27_H_22_N_4_O_2_
	II	388–710	70.69	70.81	C_6_H_5_-CH_3_, 2C_4_H_4_O-CN and C_10_H_8_N_2_	Fe; 8.92 (9.08)
[CoL(bpy)]Cl_2_·3H_2_O	I	46–84	2.91	2.88	H_2_O(hyd.)	CoC_27_H_22_N_4_O_2_Cl_2_·2H_2_O
	II	85–155	5.83	5.82	2H_2_O(hyd.)	CoC_27_H_22_N_4_O_2_Cl_2_
	III	155–357	24.74	24.63	Cl_2_, ½N_2_ and C_4_H_4_O	CoC_23_H_18_N_3_O
	IV	357–378	2.26	2.23	½N_2_	CoC_22_H_17_N_3_O
	V	379–642	48.26	48.32	2HC≡CH, C_7_H_6_ and C_10_H_8_N_2_	CoO + 2C; 16.12 (16.00)
[CuL(bpy)]Cl_2_	I	62–448	49.70	49.46	½Cl_2_, ½N_2_, C_4_H_3_CN and C_10_H_8_N_2_	CuC_12_H_11_O_2_Cl
	II	448–612	36.33	36.28	HC≡CH, C_4_H_4_O and C_6_H_5_-Cl	CuO; 14.26 (13.97)
[CdL(bpy)](NO_3_)_2_	I	63–225	4.02	4.05	HCN	CdC_26_H_21_N_5_O_8_
	II	225–413	47.74	47.70	2NO_2_, O_2_, C_3_H_4_ and C_10_H_8_N_2_	CdC_13_H_9_NO_2_
	III	413–524	25.52	24.64	C_4_H_4_O and C_6_H_5_-CN	CdO + 2C; 23.61 (22.72)

The thermal decomposition of the [FeL(bpy)]Cl_3_.H_2_O complex displayed two decomposition pathways. The first thermal breakdown occurs between 45, and 388 °C corresponds to a loss of one lattice water molecule in addition to 1½ Cl_2_ gas with estimated mass loss of 20.27% (calcd. 20.23%). The second decomposition step occurs in the 388–710 °C temperature range, coincident with the decomposition of the organic species with a mass loss of 70.81% (calcd. 70.69%) leaving Fe metal as a final residue within a weight loss of 8.92% (calc. 9.08%), and an overall mass loss equivalent to 91.08% (calcd. 90.92%).

For the [CoL(bpy)]Cl_2_.3H_2_O complex, its decomposition passed through five breakdown steps. The first, and second decomposition steps with a temperature range of 46–84, and 85–155 °C with an estimated mass loss of 2.88% (calcd. 2.91%), and 5.82% (calcd. 5.83%) resulted in the loss of one, and two lattice water molecules, respectively. The third decomposition step at (155–357 °C) due to the release of $${\raise0.7ex\hbox{$1$} \!\mathord{\left/ {\vphantom {1 2}}\right.\kern-\nulldelimiterspace} \!\lower0.7ex\hbox{$2$}}{\text{N}}_{{\text{2}}}$$, and Cl_2_ gases in addition to the furan moiety (C_4_H_4_O) with mass loss of 24.63% (calcd. 24.74%). The fourth decomposition step involved a 2.23% mass loss due to the liberation of half a mole of N_2_ gas (calcd. 2.26%). The five-step starts at 379 °C and ends at 642 °C due to the loss of 2HC≡CH, C_7_H_6_, and C_10_H_8_N_2_ with a mass loss of 48.32% (calcd. 48.26%) leaving CoO + 2C as a residue within a weight loss of 16.12% (calc. 16.00%) and an overall mass loss of 83.88% (calcd. 84.00%).

The thermal degradation of the [CuL(bpy)]Cl_2_ complex showed two stages of decomposition with a temperature range of 62–448, and 448–612 °C, respectively. The first one involved a 49.46% mass loss, which corresponds to losses of ½Cl_2_, ½N_2_, C_4_H_3_CN, and C_10_H_8_N_2_ (calcd. 49.70%). The second step specified the removal of HC≡CH, C_4_H_4_O, and C_6_H_5_-Cl fragments with a mass loss of 36.28% (calcd. 36.33%) leaving CuO residue with a weight loss of 14.26% (calcd. 13.97%) and an overall mass loss of 85.74% (calcd. 86.03%).

The thermal degradation of the [CdL(bpy)](NO_3_)_2_ complex proceeds via in three stages. The first one from 63 to 225 °C is associated with an evolution of HCN gas with a mass loss of 4.05% (calcd. 4.02%). The next breakdown stage within a temperature range 225–413 °C correlates with the elimination of two moles of NO_2_ and one mole of O_2_ gases with bipyridine, and C_3_H_4_ with a mass loss of 47.70% (calcd. 47.74%). The third decomposition phase within a range of temperature 413–524 °C corresponds to the loss of the furan ring and C_6_H_5_-CN species with a mass loss of 24.64% (calcd. 25.52%) leaving CdO + 2C residue with a weight loss of 23.61% (calcd. 22.72%) and an overall mass loss of 76.39% (calcd. 77.28%).

### Kinetic and thermodynamic study

All the synthesized mixed ligand complexes were subjected to kinetic analyses to study the kinetic, and thermodynamic parameters for their different thermal events over the studied temperature range using the formula of the Coats–Redfern (CR) Eq. (7)^53^.

7 $${\text{log}}\left[ {\frac{{{\text{log}}\left\{ {{\text{w}}_{{\text{f}}} { }/{ }\left( {{\text{w}}_{{{\text{f}} - { }}} {\text{w}}} \right)} \right\}}}{{{\text{T}}^{2} }}} \right] = {\text{log}}\left[ {\frac{{{\text{AR}}}}{{{\theta E}^{*} }}{ }\left( {1 - \frac{{2{\text{RT}}}}{{{\text{E}}^{*} }}} \right)} \right] - { }\frac{{{\text{E}}^{*} }}{{2.303{\text{RT}}}}$$

In this equation W, and W_f_ are the sample weights before degradation, and at temperature T, respectively, *A* is Arrhenius pre-exponential factor, R denotes the universal gas constant equal to 8.314 J mol^−1^ K^−1^, *θ* is the heating rate, and E* is the activation energy. Additionally, a graphical representation of this equation as given in Fig. 10b gives a straight line. The intercept of this line gives an *A* value whereas its slope gives an E* value^54^.

Besides this, the activation enthalpy (ΔH*), activation entropy (ΔS*), and activation free energy change (ΔG*) were determined by the Eyring equation^55^, and tabulated in Table 6.

**Table 6:** Thermodynamic parameters of the thermal decomposition of the mixed ligand complexes.

Mixed ligand complexes	Stage	Ts°C	Decomposition range °C	A (S^−1^)	∆H* (KJ/mol)	∆S* (KJ/mol)	∆G* (KJ/mol)	Ea (KJ/mol)	R^2^
[FeL(bpy)]Cl_3_·H_2_O	I	238	131–287	56.30 × 10^–4^	18.41	− 235.07	138.62	22.66	0.995
	II	561	510–710	2.96	68.88	− 187.04	224.83	75.82	0.995
[CoL(bpy)]Cl_2_·3H_2_O	I	107	83–148	0.82 × 10^2^	38.73	− 152.86	96.88	41.90	0.995
	II	310	288–343	18.86 × 10^3^	83.67	− 111.22	148.48	88.52	0.993
	III	397	370–465	0.44	42.38	− 201.07	177.11	47.95	0.994
	IV	578	542–610	0.12	44.57	− 213.80	226.54	51.65	0.990
[CuL(bpy)]Cl_2_	I	360	310–430	8.31 × 10	141.13	− 37.44	154.61	144.12	0.969
	II	582	430–612	1.19 × 10^9^	156.34	− 76.71	200.99	161.18	0.980
[CdL(bpy)](NO_3_)_2_	I	342	95–360	2.42 × 10^2^	20.83	− 238.75	102.51	23.68	0.981
	II	476	361–550	3.36 × 10^5^	89.05	− 143.02	157.13	93.01	0.989

The obtained data, Table 6 reveal that there is an increase in the activation energy, and the free energy change of activation values from step to step may be specified to the higher the stability of complexes, and the rigidity of the fragment resulting through the decomposition process in comparison to the original state. Furthermore, the positive ΔH, and negative ΔS values demonstrate the endothermic degradation process, and the non-spontaneous process at all^56^.

### Theoretical calculations

**Figure 11:**
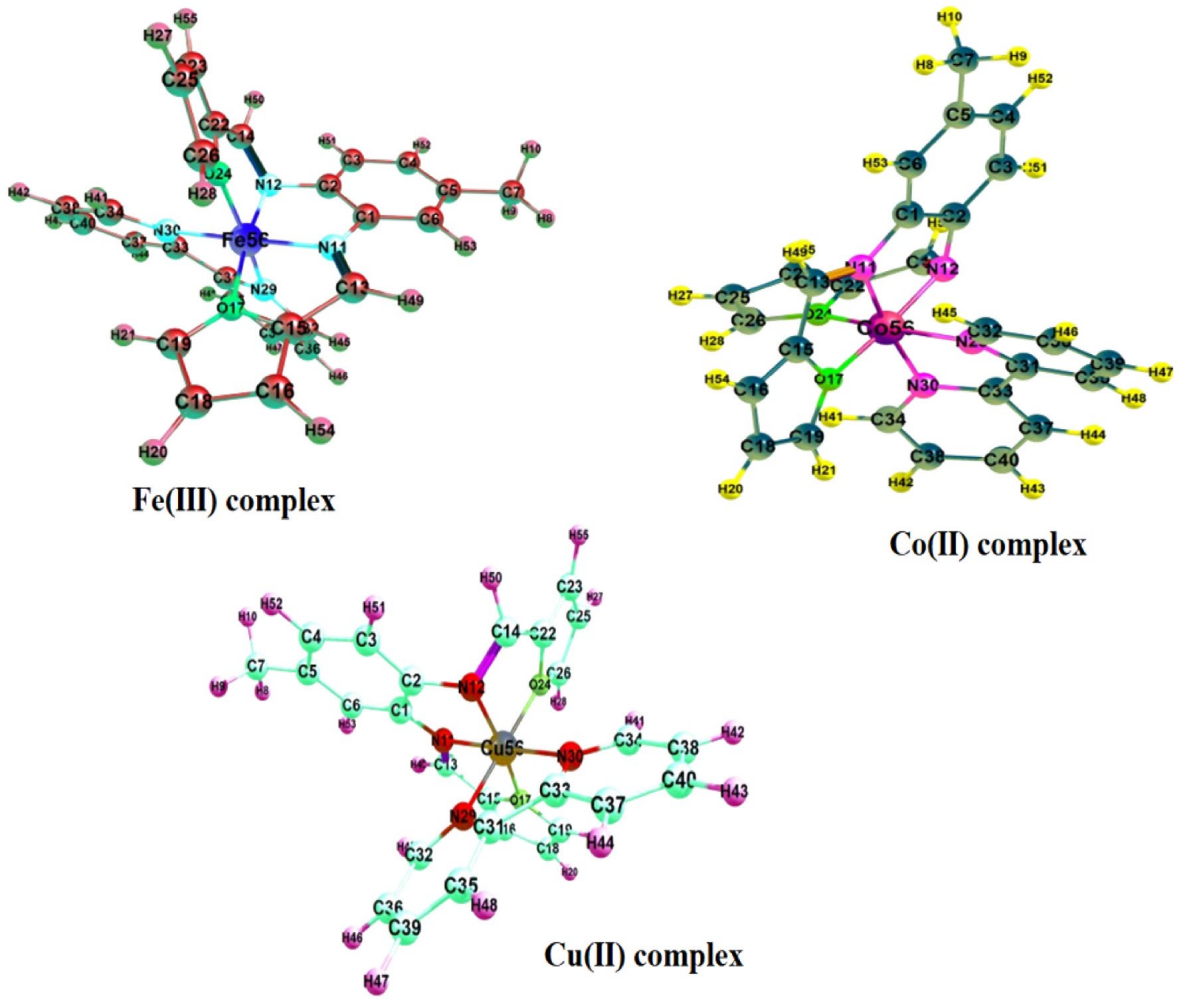
Optimized structure for the prepared mixed ligand complexes.

To investigate the three-dimensional arrangements of atoms in the mixed ligand iron(III), cobalt(II), and copper(II) complexes, molecular modelling was employed by using density functional theory (DFT) calculations. The optimized structures of the complexes reveal a distorted octahedral arrangement around the metal center, as illustrated in Fig. 11, in which the complexes have two types of coordination modes: bidentate chelating with 2,2′-bpy through both dipyridyl nitrogens (N29/N30-M), and tetradentate chelating with the imine ligand (L) through the azomethine nitrogens (N11/N12-M), and the furan oxygens (O17/O24-M). This distortion is due to the fact that the nearest coordination environment of the metal cation consists of two nitrogen atoms belong to the 2,2′-bpy co-ligand, other two nitrogen atoms belonging to the imine group, and two oxygen atoms belonging to the furan moiety. The total electronic energy, highest occupied molecular orbital energy (*E*_HOMO_), the lowest unoccupied molecular orbital energy (*E*_LUMO_), and dipole moment (*D*) were estimated for the complexes, in addition to the imine ligand (L), and the co-ligand (2,2′-bpy), and tabulated in Table 7.

**Table 7:** The calculated quantum chemical parameters for of the imine ligand (L), the co-ligand (bpy), and its mixed ligand complexes.

Molecular properties (unit)	L	bpy	[FeL(bpy)]Cl_3_·H_2_O	[CoL(bpy)]Cl_2_·3H_2_O	[CuL(bpy)]Cl_2_
E_total_ (a.u.)	− 916	− 489	− 1525	− 1549	− 1597
E_HOMO_ (eV)	− 8.190	− 8.860	− 2.721	− 1.769	− 1.769
E_LUMO_ (eV)	− 5.387	2.320	− 1.034	− 1.442	− 1.333
ΔE (E_LUMO_ − E_HOMO_) (eV)	2.803	11.08	1.687	0.327	0.436
Ionization potential IP (eV)	8.190	8.860	2.721	1.769	1.769
Electron affinity EA (eV)	5.387	− 2.320	1.034	1.442	1.333
Absolute electronegativities χ (eV)	6.788	3.270	1.878	1.605	1.551
Absolute hardness η (eV)	1.401	5.590	0.844	0.163	0.218
Absolute softness σ (eV)^−1^	0.713	0.178	1.185	6.125	4.594
Chemical potentials µ (eV)	− 6.788	− 3.270	− 1.878	− 1.605	− 1.551
Global softness S (eV)^−1^	0.356	0.089	0.593	3.062	2.297
Global electrophilicity ω (eV)	16.444	0.956	2.090	7.894	5.526
Additional electronic charge ΔN_max_	4.845	0.584	2.226	9.833	7.125
Dipole moment (Debye)	2.460	3.547	7.063	1.549	1.038

Besides, depending upon *E*_HOMO_, and* E*_LUMO_ values, additional parameters such as HOMO–LUMO gap energy (Δ*E*), chemical potential (µ), global softness (*S*), absolute hardness (η), absolute electronegativity (*χ*), absolute softness (*σ*), global electrophilicity (*ω*), electron affinity (*EA*), ionization potential (*IP*), and the additional electronic charge (Δ*N*_max_) values can be estimated via the functionally equations given below, and also tabulated in Table 7^57^.

$$\begin{aligned} \Delta E & = E_{{{\text{LUMO}}}} - E_{{{\text{HOMO}}}} ;\;\;\;\;{\rm X} = \frac{{ - \left( {E_{{{\text{HOMO}}}} + E_{{{\text{LUMO}}}} } \right)}}{2};\;\;\;\;\eta = \frac{{E_{{{\text{LUMO}}}} - E_{{{\text{HOMO}}}} }}{2};\;\;\;\;\pi = - {\rm X}; \\ \sigma & = \frac{1}{\eta };\;\;\;\;S = \frac{1}{2\eta };\;\;\;\;\omega = \frac{{\pi^{2} }}{2\eta };\;\;\;\;\Delta N_{{{\text{max}}}} = \frac{{ \pi { }}}{\eta };\;\;\;\; {\text{IP}} = - E_{{{\text{HOMO}}}} ;\;\;\;\;{\text{EA }} = - E_{{{\text{LUMO}}}} \\ \end{aligned}$$

**Figure 12:**
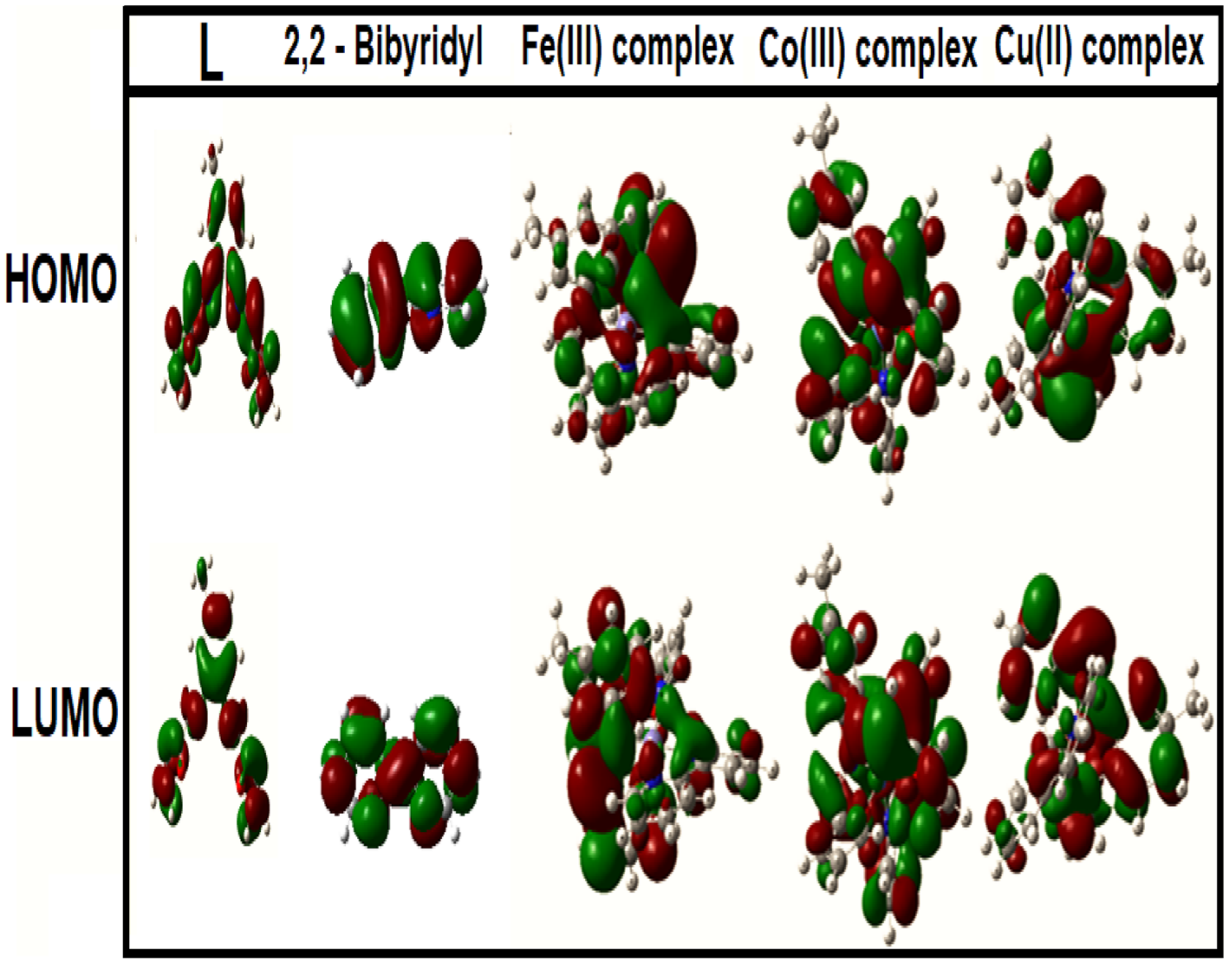
The frontier molecular orbitals of the imine ligand (L), the co-ligand (2,2′-bpy), and its mixed ligand complexes in the gas phase.

Depending upon the basic concept of the molecular orbital theory, and the data listed in Table 7, there are some important notes that can be summarized as follows:

The estimated *E*_total_ was found to be − 916 (imine ligand, L), − 489.44 (co-ligand, 2,2′-bpy), and − 1597 to − 1525 (metal complexes). The more negative values of metal complexes suggest their better stability than their parent ligands. The estimated values of the energy gap of the ligand L and co-ligand, 2,2′-bpy (ΔE = 2.803, and 11.08 eV, respectively) are greater than all synthesized complexes, so the complexes are more reactive than their parent ligands. Additionally, the [CoL(bpy)]Cl_2_·3H_2_O complex (ΔE = 0.327 eV) is the most reactive complex, while [FeL(bpy)]Cl_3_.H_2_O (ΔE = 1.687 eV) is the least reactive complex. The higher energy band gap (ΔE, 1.687 eV) of the iron (III) complex in comparison to the other complexes indicates its higher molecular chemical stability^58^. Also, among all complexes, the iron(III) complex has the highest value of the absolute hardness (η, 0.844 eV), showing it to be the chemically the hardest complex^59^. The observed trend in η values of complexes is iron(III) complex > copper(II) complex > cobalt(II) complex. Additionally, cobalt(II) complex has the highest absolute softness value (σ, 0.844 eV^−1^), with order of cobalt(II) complex > copper(II) complex > iron(III) complex. So, cobalt(II) complex is the soft one^60^. This trend is similar to the order of Δ*E* where, the hard molecules possess large energy gap values whereas, the soft molecules possess small energy gap values^61^. The higher value of the ionization potential (*IP*, 2.721 eV) of the iron(III) complex confirms its higher stability in comparison to other complexes^62^. Figure 12 shows that the electron densities of HOMO, and LUMO are localized on the metal, and coordinated atoms of ligands.

### Antimicrobial evaluation

**Figure 13:**
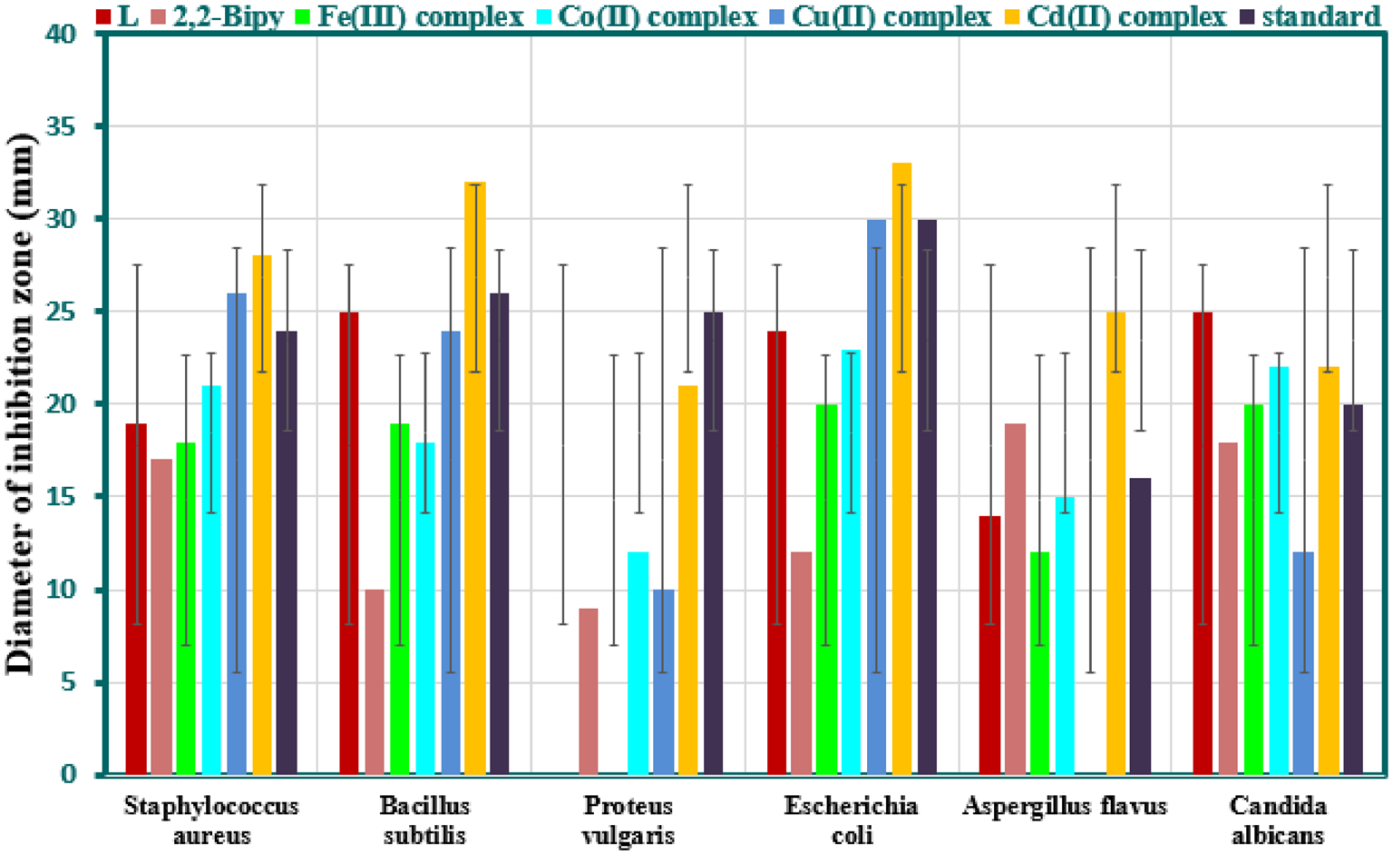
Antimicrobial activity of the imine ligand (L), the co-ligand (2,2′-bpy), and mixed ligand complexes.

All synthesized mixed ligand complexes in addition to the imine ligand (L), and co-ligand 2,2′-bpy were tested for in vitro antimicrobial activity against different species of bacterial, and fungal strains using the method of paper disk diffusion and measuring the relevant inhibition zone value (IZV) as mentioned in the experimental part. The results obtained in addition to the estimated activity index (AI) were tabulated in Table 8, and depicted in Fig. 13. It is clear from Table 8, the observed results varied in terms of IZV, and AI, and generally the imine ligand (L), co-ligand (2,2′-bpy), and all mixed ligand complexes exhibited a variable degree of antimicrobial activity against the selected bacterial, and fungal pathogens. According to these results, some remarkable points were summarized for bacterial and fungal assays.

**Table 8:** Antimicrobial assay of the Schiff-base ligand and its mixed ligand complexes.

Mixed ligand complexes	Zones diameter showing complete growth inhibition (mm)					
	Gram-positive bacteria		Gram-negative bacteria		Fungi	
	*S. aureus*	*B. subtilis*	*P. vulgaris*	*E. coli*	*A. flavus*	*C. albicans*
[FeL(bpy)]Cl_3_·H_2_O	18 (75)	19 (73)	NA	20 (67)	12 (75)	20 (100)
[CoL(bpy)]Cl_2_·3H_2_O	21 (88)	18 (69)	12 (48)	23 (77)	15 (94)	22 (110)
[CuL(bpy)]Cl_2_	26 (108)	24 (92)	10 (40)	30 (100)	NA	12 (60)
[CdL(bpy)](NO_3_)_2_	28 (117)	32 (124)	21 (84)	33(110)	25 (156)	22 (110)
L	19 (79)	25 (96)	NA	24 (80)	14 (88)	25 (125)
2,2′-Bipy	17(70.83)	10(83.46)	9(36)	12(40)	19(118.75)	18(90)
*Gentamycin*	24	26	25	30	–	–
*Ketoconazole*	–	–	–	–	16	20
DMSO	0	0	0	0	0	0

#### Bacterial inhibition assay

- The imine ligand (L) showed antibacterial action against all tested organisms except toward *P. vulgaris*, it showed no sensitivity. Also, it displays an IZV of 25 mm against *B. subtilis* which is comparable to the standard *gentamycin* value (26 mm).
- The co-ligand (2,2′-bpy) showed antibacterial activity against all tested organisms that was lower than imine ligand (L) except toward *P. vulgaris*.
- For all mixed ligand complexes, each one exhibited a clear, and distinct activity against the bacterial pathogens. Among all mixed ligand complexes, no microbial growth inhibition was observed with [FeL(bpy)]Cl_3_.H_2_O complex only toward *P. vulgaris*.
- [CdL(bpy)](NO_3_)_2_ displayed higher influences against all pathogenic bacteria as compared to the other complexes, the imine ligand (L), and the co-ligand (2,2′-bpy). Additionally, the inhibitory activity of this complex against the mentioned organisms was found to be higher than that of the standards e*xcept P. vulgaris*.
- The inhibitory activity of [CuL(bpy)]Cl_2_ complex with an IZV of 26 mm against *S. aureus* was found to be higher than that of the *Gentamycin* standard (24 mm), [CoL(bpy)]Cl_2_·3H_2_O (21 mm), and [FeL(bpy)]Cl_3_.H_2_O (18 mm). The order of activity for all tested compounds was found to be: [CdL(bpy)](NO_3_)_2_ > [CuL(bpy)]Cl_2_, > *Gentamycin* standard > [CoL(bpy)]Cl_2_·3H_2_O > (L) > [FeL(bpy)]Cl_3_.H_2_O > (2,2′-bpy).
- The efficacy of the [CuL(bpy)]Cl_2_ complex (30 mm) was found to be equal to that of the *Gentamycin* standard (30 mm) against *E. coli*.
- It is clear that, *E. Coli* is inhibited by all mixed ligand complexes more than *S. aureus*, *B. subtilis*, and *P. vulgaris.* Similarly, *P. vulgaris* is inhibited by all mixed ligand complexes less than to *E. coli*, *S. aureus*, and *B. subtilis*. This is an indication to sensitivity of these mixed ligand complexes, and fairly can be applied in treatment of certain diseases caused by certain organism than others.

#### Fungal inhibition assay

- The imine ligand (L), and co-ligand (2,2′-bpy) showed remarkable antifungal action against *A. flavus*, and *C. albicans* but to different degrees. The imine ligand (L) showed better activity with an IZV of 25 mm against *C. albicans*. This value is higher than the co-ligand (2,2′-Bipy) (18 mm), and the standard *Ketoconazole* value (20 mm). Moreover, the co-ligand (2,2′-bpy) showed higher action against *A. flavus*. It displays an IZV of 19 mm in comparison to imine ligand (L) (14 mm), and the standard *Ketoconazole* (16 mm).
- The results of the inhibition of fungal species growth showed that the highest activity was noted for [CdL(bpy)](NO_3_)_2_ against *A. flavus* with an IZV of 25 mm, higher than *Ketoconazole* standard (16 mm) and other metal complexes. The order of inhibition activities for all tested compounds recorded for *A. flavus* is: CdL(bpy)](NO_3_)_2_ > 2,2′-bpy > *Ketoconazole* > [CoL(bpy)]Cl_2_·3H_2_O > L > [FeL(bpy)]Cl_3_.H_2_O.
- Both [CoL(bpy)]Cl_2_.3H_2_O, and [CdL(bpy)](NO_3_)_2_ complexes exhibited equal inhibitory effects against *C. albicans* with IZV of 22 mm, and significantly higher than *Ketoconazole* standard (20 mm). Furthermore, [FeL(bpy)]Cl_3_.H_2_O complex has equal activity (20 mm) to *Ketoconazole* standard against the same organism. The order of inhibition activities for all tested compounds recorded for *C. albicans* as: L > [CoL(bpy)]Cl_2_.3H_2_O = [CdL(bpy)](NO_3_)_2_ > *Ketoconazole* = [FeL(bpy)]Cl_3_.H_2_O > 2,2′-bpy > [CuL(bpy)]Cl_2_.
- Among all mixed ligand complexes, the [CuL(bpy)]Cl_2_ complex showed no fungal growth inhibition towards *A. flavus*.

Finally, as per the literature review, the diversity in the effectiveness of the tested compounds towards the bacteria, and fungi species can be related to the easy penetration, and more interference of the sample into the cell wall. This is based on different factors such as cell membrane, cell permeability, and disruption of the cytoplasmic membrane as a result of protein synthesis inhibition. Also, metal ion nature, donor site nature, and metal complex formation have great effect as described through Tweedy’s chelation theory. The easy penetration, more interference of the complex into the cell through H-bond formation are the concepts of this theory cells^63–65^.

## Conclusion

In the present paper, four new mononuclear mixed ligand Fe(III), Co(II), Cu(II), and Cd(II) complexes have been successfully prepared using the furfural-type imine ligand (L), and the co-ligand 2,2′-bipyridine (2,2′-bpy), and structurally characterized using different techniques. Correlation of all obtained data confirmed their suggested composition, and structure. The aim to synthesis such complexes is the combination of different bioactive molecules in addition to incorporation some metal ions as Fe(III), Co(II), Cu(II), and Cd(II) forming significant complexes as antimicrobial agents characterize with enhanced antimicrobial activity. All the synthesized mixed ligand complexes in comparison to their parent ligands; the imine ligand (L), and co-ligand (2,2′-bpy) were subject to study their inhibitor activity toward some bacterial, and fungi strains. The novelty of this research article is the screening results of the biological evaluation of these complexes indicating the sensitivity of some of these mixed ligand complexes toward some pathogenic under study in addition to the higher action than the standard antibiotic. This outcome provides a type of complexes of medicinal value consider as an attractive target for antimicrobial drug development.

## Data Availability

The data used to support the findings of this study are included in the article.

## References

[1] Bouhdada M, El Amane M, El Hamdani H, Khiya Z (2023). Synthesis, characterization, biological evaluation and molecular docking studies of salicylidene-aniline and their metal mixed ligand complexes with caffeine. J. Mol. Struct..

[2] Chethan BS, Rajegowda HR, Khan RR, Lokanath NK (2023). Structural and theoretical insights towards the understanding of the effect on the conformation of ligand by complexation process. J. Mol. Struct..

[3] Kaur P, Verma I, Khanum G, Siddiqui N, Javed S, Arora H (2023). Dimeric Zn II complex of carboxylate-appended (2-pyridyl)alkylamine ligand and exploration of experimental, theoretical, molecular docking and electronic excitation studies of ligand. J. Mol. Struct..

[4] Abd El-Wahab ZH, Mashaly MM, Salman AA, El-Shetary BA, Faheim AA (2004). Co(II), Ce(III) and UO_2_(VI) bis-salicylatothiosemicarbazide complexes Binary and ternary complexes, thermal studies and antimicrobial activity. Spectrochim. Acta A..

[5] Mashaly MM, Abd El-Wahab ZH, Faheim AA (2004). Mixed-ligand complexes of a schiff base, 8-hydroxyquinoline and oxalic acid with Cu(II), Ni(II), Zn(II), and Fe(III) Ions: Pyrolytic products and biological activities. Synth. React. Inorg. Met.-Org. Chem..

[6] Mashaly MM, Abd El-Wahab ZH, Faheim AA (2004). Preparation, spectral characterization and antimicrobial activities of schiff base complexes derived from 4-aminoantipyrine. Mixed ligand complexes with 2-aminopyridine, 8-hydroxyquinoline and oxalic acid and their pyrolytical products. J. Chin. Chem. Soc..

[7] Abd El-Wahab ZH, Mashaly MM, Faheim AA (2005). Synthesis and characterization of cobalt(II), cerium(III), and dioxouranium(VI) complexes of 2,3-dimethyl-1-phenyl 4-salicylidene-3-pyrazolin-5-one mixed ligand complexes, pyrolytic products, and biological activities. Chem. Paper.

[8] Mohamed GG, Abd El-Wahab ZH (2005). Mixed ligand complexes of bis(phenylimine) Schiff base ligands incorporating pyridinium moiety Synthesis, characterization and antibacterial activity. Spectrochim. Acta A.

[9] Abd El-Wahab ZH (2008). Mixed ligand complexes of nickel(II) and cerium(III) ions with 4-(-3 methoxy-4-hydroxybenzylideneamino)-1, 3-dimethyl-2,6-pyrimidine-dione and some nitrogen/oxygen donor ligands. J. Coord. Chem..

[10] El-Shafiey ZA, Abd El-Wahab ZH, Salman AA, Taha RH (2010). Study of binary and ternary complexes of Co(II), Ni(II), Cd(II), Fe(III), and UO_2_(II) complexes of amino carboxylic acid derivatives and pyridine, synthesis, spectroscopic characterization, thermal investigation and biological activity. Egypt. J. Chem..

[11] Ibrahim AA, Adel AM, Abd-El-Wahab ZH, Al-Shemy MT (2011). Utilization of carboxymethyl cellulose based on bean hulls as chelating agent. Synthesis, characterization and biological activity. Carbohydr. Poly..

[12] Faheim AA, Abdou SN, Abd El-Wahab ZH (2013). Synthesis and characterization of binary and ternary complexes of Co(II), Ni(II), Cu(II) and Zn(II) ions based on 4-aminotoluene-3-sulfonic acid. Spectrochim. Acta A.

[13] Feng X, Ren Y, Jiang H (2021). Metal-bipyridine/phenanthroline-functionalized porous crystalline materials: Synthesis and catalysis. Coord. Chem. Rev..

[14] Jara Y, Araujo ML, Madden W, Lubes V, Hernandez L (2022). Ternary complex formation of the nickel(II), 2,2’-bipyridine, 1,10’-Phenanthroline and some amino acids. Phys. Chem. Liq..

[15] Masoud MS, Soayed AA, Almesmari SA, Elsamra RMI (2021). New mixed-ligand complexes of cytosine and its silver nanoparticles: Spectral, analytical, theoretical and biological activity studies. J. Inorg. Organomet. Poly. Mater..

[16] Ismail BA, Nassar DA, Abd El-Wahab ZH, Ali OAM (2021). Synthesis, characterization, thermal, DFT computational studies and anticancer activity of furfural-type Schiff base complexes. J. Mol. Struct..

[17] Vogel AI. *A Text Book of Quantitative Inorganic Analysis* third edn, Longman ELBS, London, 5th edn (John Wiley and Sons, Inc. New York, 1989).

[18] Khalf-Alla PA, Hassan SS, Shoukry MM (2019). Complex formation equilibria, DFT, docking, antioxidant and antimicrobial studies of iron(III) complexes involving Schiff bases derived from glucosamine or ethanolamine. Inorg. Chim. Acta.

[19] Andiappan K, Sanmugam A, Deivanayagam E, Karuppasamy K, Kim HS, Vikraman D (2019). Schiff base rare earth metal complexes: Studies on functional, optical and thermal properties and assessment of antibacterial activity. Int. J. Biol. Macromol..

[20] Rahaman F, Gupta P, Manjunatha MN, Gautam P (2022). Benzo [g] indole-based Schiff’s base ligand and its transition metal complexes: Synthesis, characterization and anti-microbial activity studies. Mater. Today Proc..

[21] Biernacka IK, Raposo MMM, Batista RMF, Soares OSGP, Pereira MFR, Parpot P, Oliveira C, Skiba E, Jartych E, Fonseca AM, Neves IC (2020). Binuclear furanyl-azine metal complexes encapsulated in NaY zeolite as efficiently heterogeneous catalysts for phenol hydroxylation. J. Mole. Struct..

[22] Salem AE, Mohammed SF, Sadeek SA, Zordok WA, El-Attar MS (2022). Synthesis, structural elucidation, molecular modeling, andantimicrobial studies of some nanoparticles mixed ligandscomplexes of cetirizine in presence of 2,20-bipyridine. Appl. Organomet. Chem..

[23] Ajibola AA, Grice KA, Perveen F, Wojciechowska A, Sieron L, Maniukiewicz W (2021). Synthesis, crystal structures, Hirshfeld surface analysis, theoretical insight and molecular docking studies of dinuclear and triply bridged Cu(II) carboxylate complexes with 2,2′-bipyridine or 1,10-phenanthroline. Polyhedron.

[24] Kresakova L, Mino A, Holub M, Kuchar J, Werner A, Tomas M, Cizmar E, Falvello LR, Cernak J (2021). Heteroleptic complexes of Ni(II) with 2,2′-bipyridine and benzoato ligands. Magnetic properties of [Ni(bpy)(Bz)2]. Inorg. Chim. Acta.

[25] Gelik S, Yurdakul S, Erdem B (2021). Synthesis, spectroscopic characterization (FT-IR, PL), DFT calculations and antibacterial activity of silver(I) nitrate complex with nicotinaldehyde. Inorg. Chem. Commun..

[26] Kargar H, Mehrjardi MF, Ardakani RB, Munawar KS (2021). Synthesis, spectra (FT-IR, NMR) investigations, DFT, FMO, MEP, NBO analysis and catalytic activity of MoO_2_(VI) complex with ONO tridentate hydrazone Schiff base ligand. J. Mol. Struct..

[27] Aldulmani SAA (2021). Spectral, modeling, dna binding/cleavage and biological activity studies on the newly synthesized 4-[(Furan-2-ylmethylene)amino]-3-[(2–hydroxy–benzylidene)amino]-phenyl}-phenyl-methanone and some bivalent metal(II) chelates. J. Mol. Struct..

[28] Kothandan S, Sheela A (2020). Design of oxoperoxovanadium(V) complexes and their DNA interaction studies. J. Coord. Chem..

[29] Saedi Z, Hoveizi E, Roushani M, Massahi S, Hadian M, Salehi K (2019). Synthesis, characterization, anticancer properties and theoretical study of asymmetrical Cd(II)eN_2_-Schiff base complexes. J. Mol. Struct..

[30] Ali I, Wani WA, Saleem K (2013). Empirical formula to molecular structures of metal complexes by molar conductance. Synth. React. Inorg. Met. Org. Nano-Met. Chem..

[31] Amin BH, Abou-Dobara MI, Diab MA, Gomaa EA, El-Mogazy MA, El-Sonbati AZ, El-Ghareib MS, Hussien MA, Salama HM (2020). Synthesis, characterization, and biological investigation of new mixed-ligand complexes. Appl. Organomet. Chem..

[32] Smekal Z, Novak P, Zeller M, Antal P, Cizmar E, Herchel R (2022). Synthesis, crystal structure, 57Fe Mossbauer ¨ spectroscopy and magnetic properties of high-spin iron(III) anionic complexes [Fe(azp)2]- (H2azp = 2,2′- dihydroxyazobenzene) with organic cations. Polyhedron.

[33] Abou-Melha KS, Al-Hazmi GA, Althagafi I, Alharbi A, Shaaban F, El-Metwaly NM, El-Bindary AA, El-Bindary MA (2021). synthesis, characterization, DFT calculation, DNA binding and antimicrobial activities of metal complexes of dimedone arylhydrazone. J. Mol. Liq..

[34] Patil SK, Vibhute BT (2021). Synthesis, characterization, anticancer and DNA photocleavage study of novel quinoline Schiff base and its metal complexes. Arab. J. Chem..

[35] Sennappan M, Krishna PM, Managutti PB, Mangasuli SN, Malini S (2021). Nucleic acid Interaction and photoluminescent properties of acylhydrazone and Its Mn(II), Co(II), Cu(II), Zn(II) and Cd(II) complexes. Chem. Afr..

[36] Tofiq DI, Hassan HQ, Abdalkarim KA (2021). Preparation of a novel Mixed-Ligand divalent metal complexes from solvent free synthesized Schiff base derived from 2,6-diaminopyridine with cinnamaldehyde and 2,20-Bipyridine: Characterization and antibacterial activities. Arab. J. Chem..

[37] Sundaram GA, Vaithinathan K, Anbalagan K (2021). New monomeric mixed-ligand complex of iron(III)-3-chloropyridine: Synthesis, structure, luminescence, electrochemical and magnetic properties. J. Mol. Struct..

[38] El-ghamry MA, Nassir KM, Elzawawi FM, Abdel Aziz AA, Abu-El-Wafa SM (2021). Novel nanoparticle-size metal complexes derived from acyclovir. Spectroscopic characterization, thermal analysis, antitumor screening, and DNA cleavage, as well as 3D modeling, docking, and electrical conductivity studies. J. Mol. Struct..

[39] Abd El-Wahab ZH (2008). Complexation of 4-amino-1,3 dimethyl-2,6 pyrimidine-dione derivatives with cobalt(II) and nickel(II) ions: Synthesis, spectral, thermal and antimicrobial studies. J. Coord. Chem..

[40] Elsawy MM, Faheim AA, Salem SS, Owda ME, Abd El-Wahab ZH, Abd El-Wahab H (2021). Cu (II), Zn (II), and Ce (III) metal complexes as antimicrobial pigments for surface coating and flexographic ink. Appl. Organomet. Chem..

[41] Attri P, Garg S, Ratan JK (2021). Kinetic modelling and proposed mechanistic pathway for photocatalytic degradation of 4-aminopyridine using cuprous oxide nanoparticles. Res. Chem. Intermed..

[42] Evingur GA, Pekcan O (2018). Optical energy band gap of PAAm-GO composites. Compos. Struct..

[43] Dgachi S, Rahmouni F, Soran A, Saoudi M, Nemes G, Naili H (2021). A mononuclear Co(II) complex: Crystal structure, thermal behavior, optical properties and biological activities. J. Mol. Struct..

[44] Ali HE, Abdel-Aziz MM, Algarni H, Yahia IS, Khairy Y (2021). Multifunctional applications of a novel Ru-metal mixed PVAL flexible composite for limiting absorption and varistor: Synthesis, optical, and electrical characterization. J. Inorg. Organomet. Poly. Mater..

[45] Triki H, Nagy B, Overgaard J, Jensen F, Kamoun S (2020). Structure, DFT based investigations on vibrational and nonlinear optical behavior of a new guanidinium cobalt thiocyanate complex. Struct. Chem..

[46] El-Gammal OA, Saad DA, Al-Hossainy AF (2021). Synthesis, spectral characterization, optical properties and x-ray structural studies of S centrosymmetric N_2_S_2_ or N_2_S_2_O_2_ donor Schiff base ligand and its binuclear transition metal complexes. J. Mol. Struct..

[47] Rao PN, Reddy MCS, Reddy AP, Kumar ER, Rao BA (2021). Optical and dielectric studies of CdI2-doped silver borotelurate glass system. J. Mater. Sci. Mater. Electron..

[48] Ningthemcha RKN, Mondal R, Das AS, Debnath S, Kabi S, Singh LS, Biswas D (2022). The effect of transition metal and heavy metal incorporation on the structural, optical and electrical properties of zinc-phosphate ternary glassy system: A comparative study. Mater. Chem. Phy..

[49] Khanagwal J, Khatkar SP, Dhankhar P, Bala M, Kumar R, Boora P, Taxak VB (2020). Synthesis and photoluminescence analysis of europium(III) complexes with pyrazole acid and nitrogen containing auxiliary ligands. Spectrosc. Lett. Intern. J. Rapid Commun..

[50] Zhukovsky M, Koubisy MSI, Zakaly HMH, Ali AS, Issa SAM, Tekin HO (2022). Dielectric, structural, optical and radiation shielding properties of newly synthesized CaO–SiO_2_–Na_2_O–Al_2_O_3_ glasses: Experimental and theoretical investigations on impact of Tungsten(III) oxide. Appl. Phys. A.

[51] Mohammed MI, Bouzidi A, Zahran HY, Jalalah M, Harraz FA, Yahia IS (2021). Ammonium iodide salt-doped polyvinyl alcohol polymeric electrolyte for UV-shielding filters: Synthesis, optical and dielectric characteristics. J. Mater. Sci. Mater Electron..

[52] Dhanaraj CJ, Raj SSS (2020). Synthesis, characterization and biological studies of S chiff base metalcomplexes derived from 4-aminoantipyrine, acetamide and pphenylenediamine. Inorg. Chem. Commun..

[53] Abu-Dief AM, El-Metwaly NM, Alzahrani SO, Alkhatib F, Abumelha HM, El-Dabea T, El-Remaily MAAA (2021). Structural, conformational and therapeutic studies on new thiazole complexes: Drug-likeness and MOE-simulation assessments. Res. Chem. Intermed..

[54] Al-Hazmi GAA, Bou-Melha KS, El-Metwaly NM, Althagaf I, Zaki R, Shaaban F (2020). Green synthesis for 3-(2-Benzoylhydrazono)-*N*-(pyridin-2-yl) butanamide complexes: Spectral, analytical, modelling, MOE docking and biological studies. J. Inorg. Organomet. Poly. Mater..

[55] El-Gammal OA, Mohamed FS, Rezk GN, El-Bindary AA (2021). Structural characterization and biological activity of a new metal complexes based of Schiff base. J. Mol. Liq..

[56] Shah R, Alharbi A, Hameed AM, Saad F, Zaky R, Khedr AM, El-Metwaly N (2020). Synthesis and structural elucidation for new schiff base complexes; Conductance, conformational, MOE-docking and biological studies. J. Inorg. Organomet. Poly. Mater..

[57] Patel AK, Patel N, Patel RN, Jadeja RN (2022). New copper(II) μ-alkoxo-μ-carboxylato double-bridged complexes as models for the active site of catechol oxidase: Synthesis, spectral characterization and DFT calculations. Heliyon.

[58] Singh A, Gogoi HP, Barman P (2022). Comparative study of palladium(II) complexes bearing tridentate ONS and NNS Schiff base ligands: Synthesis, characterization, DFT calculation, DNA binding, bioactivities, catalytic activity, and molecular docking. Polyhedron.

[59] Sumrra SH, Zafar W, Asghar ML, Mushtaq F, Raza MA, Nazar MF, Nadeem MA, Imran M, Mumtaz S (2021). Computational investigation of molecular structures, spectroscopic properties, cholinesterase inhibition and antibacterial activities of triazole Schiffbases endowed metal chelates. J. Mol. Struct..

[60] Korkmaz Ü, Findik BT, Dede B, Karipcin F (2022). Synthesis, structural elucidation, invitro antibacterial activity, DFT calculations, and molecular docking aspects of mixed-ligand complexes of a novel oxime and phenylalanine. Bioorg. Chem..

[61] Parvarinezhad S, Salehi M, Kubicki M (2022). Synthesis, characterization, spectral studies and evaluation of noncovalent interactions in co-crystal of μ-oxobridged polymeric copper(II) complex derived from pyrazolone by theoretical studies. J. Mol. Struct..

[62] Waheeb AS, Kyhoiesh HAK, Salman AW, Al-Adilee KJ, Kadhim MM (2022). Metal complexes of a new azo ligand 2-[2′-(5-nitrothiazolyl) azo]-4-methoxyphenol (NTAMP): Synthesis, spectral characterization, and theoretical calculation. Inorg. Chem. Commun..

[63] Sumrra SH, Sahrish I, Raza MA, Ahmad Z, Zafar MN, Chohan ZH, Khalid M, Ahmed S (2020). Efcient synthesis, characterization, and in vitro bactericidal studies of unsymmetrically substituted triazole-derived Schiff base ligand and its transition metal complexes. Monatsh. für Chem..

[64] Beyene BB, Mihirteu AM, Ayana MT, Yibeltal AW (2020). Synthesis, characterization and antibacterial activity of metalloporphyrins: Role of central metal ion. Results Chem..

[65] Chaudhary NK, Guragain B, Chaudhary A, Chaudhary SK (2021). Heteroleptic cadmium complex of glimepiride–metformin mixed ligand: synthesis, characterization, and antibacterial study. Chem. Pap..

